# Identification of a dCache-type chemoreceptor in *Campylobacter jejuni* that specifically mediates chemotaxis towards methyl pyruvate

**DOI:** 10.3389/fmicb.2024.1400284

**Published:** 2024-05-09

**Authors:** Qi Zhao, Fulian Yao, Wei Li, Shuangjiang Liu, Shuangyu Bi

**Affiliations:** ^1^State Key Laboratory of Microbial Technology, Shandong University, Qingdao, China; ^2^Department of Clinical Laboratory, Qilu Hospital, Shandong University, Jinan, China; ^3^State Key Laboratory of Microbial Resources, and Environmental Microbiology Research Center, Institute of Microbiology, Chinese Academy of Sciences, Beijing, China

**Keywords:** chemoreceptor Tlp11, chemotaxis, *Campylobacter jejuni*, ligands, chimeras

## Abstract

The foodborne pathogenic bacterium *Campylobacter jejuni* utilizes chemotaxis to assist in the colonization of host niches. A key to revealing the relationship among chemotaxis and pathogenicity is the discovery of signaling molecules perceived by the chemoreceptors. The *C. jejuni* chemoreceptor Tlp11 is encoded by the highly infective *C. jejuni* strains. In the present study, we report that the dCache-type ligand-binding domain (LBD) of *C. jejuni* ATCC 33560 Tlp11 binds directly to novel ligands methyl pyruvate, toluene, and quinoline using the same pocket. Methyl pyruvate elicits a strong chemoattractant response, while toluene and quinoline function as the antagonists without triggering chemotaxis. The sensory LBD was used to control heterologous proteins by constructing chimeras, indicating that the signal induced by methyl pyruvate is transmitted across the membrane. In addition, bioinformatics and experiments revealed that the dCache domains with methyl pyruvate-binding sites and ability are widely distributed in the order Campylobacterales. This is the first report to identify the class of dCache chemoreceptors that bind to attractant methyl pyruvate and antagonists toluene and quinoline. Our research provides a foundation for understanding the chemotaxis and virulence of *C. jejuni* and lays a basis for the control of this foodborne pathogen.

## 1 Introduction

*Campylobacter jejuni*, as a zoonotic pathogen, can cause acute human gastrointestinal (GI) diseases and serious complications, including meningitis, urinary tract infection, and Guillain-Barre syndrome (Young et al., 2007; O’brien, 2017). Approximately 10% of the world’s population suffers from campylobacteriosis (Elgamoudi et al., 2021). *C. jejuni* is listed as one of the Top 10 pathogenic bacteria that cause foodborne diseases. To find a suitable environment for growth and overcome challenges in hosts, *C. jejuni* can respond rapidly to external stimuli through its signal transduction cascades (Chandrashekhar et al., 2017). Studies on the mechanisms by which *C. jejuni* cells sense environmental signals and adapt to living niches are crucial for understanding their colonization and pathogenic processes.

The chemotaxis network, as a primary chemosensory system, enables motile bacteria or archaea to navigate the gradients of signal molecules, including attractants and repellents, to find optimal niches for growth (Colin et al., 2021). Increasing evidence suggests that chemotaxis and motility are crucial virulence factors for colonization, initial infection, and post-inflammatory stages of *C. jejuni* and other GI tract-inhabiting pathogens (Wassenaar et al., 1993; Yao et al., 1997; Matilla and Krell, 2018; Korolik, 2019; Zhou et al., 2023). The components and signal transduction mechanisms of chemotaxis networks are highly conserved among chemotactic bacteria (Wuichet and Zhulin, 2010). Chemoreceptors (also termed methyl-accepting chemotaxis proteins or transducer-like proteins [Tlps]) typically oligomerize into trimers of dimers, to form repetitive hexagonal arrays, sense stimuli, and transmit signals to regulate the activity of the histidine kinase CheA (Hazelbauer et al., 2008; Briegel et al., 2012; Riechmann and Zhang, 2023). The response regulator CheY accepts a phosphoryl group from the activated CheA, while the phosphorylated CheY interacts with the flagellar motor(s), thereby changing the direction of flagellar rotation and allowing bacteria to swim towards attractants or away from repellents (Bi and Sourjik, 2018). In addition, the methyltransferase CheR- or methylesterase CheB-dependent adaptation rate is slower than the time scale of signal transmission, thereby providing the cells short-term memory, to compare the current environment with the conditions encountered in the past few seconds (Kim et al., 2002; Parkinson et al., 2015).

It has been reported that *C. jejuni* strains encode 13 putative chemoreceptors, with Tlp1, Tlp2, Tlp3, Tlp4, Tlp7, and Tlp10 present in most *C. jejuni* strains (Day et al., 2012; Mund et al., 2016), and other chemoreceptors, such as Tlp11, Tlp12, and Tlp13, present in only some isolates (Day et al., 2016; Mund et al., 2016). Previous reports have shown that Tlp11 is the least common chemoreceptor in *C. jejuni* (Day et al., 2012), and approximately 11% of isolates from humans and chickens encode it (Day et al., 2016). Moreover, according to receptor topology, Tlp1-Tlp4, Tlp7, and Tlp10-Tlp13 belong to the Class I group (Marchant et al., 2002; Korolik, 2019), which contains a periplasmic ligand-binding domain (LBD), two transmembrane helices, 1–2 Histidine kinases, Adenyl cyclases, Methyl-accepting chemotaxis proteins and Phosphatases (HAMP) domain(s), and a methyl-accepting transducer domain. In the conventional sensing mechanism, a signal molecule binds directly to the ligand-binding pocket of an LBD (Bi and Sourjik, 2018). The discovery of direct-binding ligands of chemoreceptors is key to understanding the physiological role of chemotactic behavior. However, except for several chemoreceptors (Day et al., 2016; Khan et al., 2020; Elgamoudi et al., 2021; Taha et al., 2022; Duan et al., 2023), there remain gaps in the identification of *C. jejuni* chemoreceptor ligands and understanding of their functions.

Tlp11-encoding *C. jejuni* strains are mainly derived from those that cause human campylobacteriosis (Day et al., 2012). A research on the evolutionary origin of Tlp11 showed that it only appears in a few highly virulent strains; therefore, the chemoreceptor Tlp11 may be a marker of virulence in *C. jejuni* (Day et al., 2016). Indeed, *C. jejuni* strains containing Tlp11 exhibit high autoagglutination ability, which is involved in the formation of biofilms, thereby enhancing the virulence of *C. jejuni* (Guerry et al., 2006; Day et al., 2016). In a chicken colonization model, upon oral administration of *C. jejuni* 520 wild-type (WT) and 520/ΔTlp11 strains to chicken, the inactivated Tlp11 in *C. jejuni* showed a significant reduction in colonization of ceca, as compared to the WT. In addition, the expression of Tlp11 resulted in higher adhesion to polarized Caco-2 and HCT 116 cells, as compared to that observed in *C. jejuni* strains that do no not express Tlp11 (Day et al., 2016). Therefore, identifying a full set of ligands for Tlp11 and characterizing its physiological function are crucial for understanding the interactions of *C. jejuni* with its hosts, through chemotaxis.

Tlp11-LBD has been reported as a double Calcium channels and chemotaxis (dCache) domain (Day et al., 2016). The dCache domain belongs to the Cache-like superfamily, which is the largest group of sensory domains in bacteria (Ortega et al., 2017) and can sense various types of ligands (Ortega et al., 2017; Matilla et al., 2022). It contains membrane-proximal and -distal subdomains, both of which may contain ligand-binding pockets. In case of most of the reported dCache domains, ligands bind to the membrane-distal subdomain (Gavira et al., 2018; Khan et al., 2020), whereas a few studies have suggested that both subdomains might bind ligands (Machuca et al., 2017; Johnson et al., 2021; Feng et al., 2022). A previous study suggested that Tlp11-LBD of *C. jejuni* bound directly to galactose. However, galactose could not be metabolized by *C. jejuni* (Day et al., 2016). As obtaining nutrients is the main selective force leading to chemotactic evolution (Matilla et al., 2023a), identifying a signaling molecule of Tlp11 that has metabolic value for *C. jejuni* is of great interest.

Exploring whether ligand binding to a specific receptor, the LBD, can transmit signals is crucial for revealing the physiological function of the ligand. The construction of chimeras is a potential tool to elucidate the ligand specificities and signaling properties of target receptor LBDs (Bi et al., 2016; Luu et al., 2019; Duan et al., 2023). Because some transmembrane chemoreceptors and histidine kinases have similar topologies, the target LBD can be fused with a chemoreceptor or histidine kinase to form a hybrid chemoreceptor or hybrid kinase. The signaling properties of the target LBD in response to the ligand could thus be characterized using standard chemotaxis assays and model bacteria, such as measuring the chemotactic responses of *Escherichia coli* expressing the hybrid chemoreceptor using microfluidics or fluorescence resonance energy transfer (FRET) assays (Sourjik et al., 2007), or verified by means of detection of fluorescence signals using a fluorescent reporter gene that is placed under the promoter controlled by the hybrid kinase and corresponding response regulator. These chimera construction strategies not only allow ligand recognition and functional verification of the target LBD on signal transmission, but also allow quantification of the relative affinity for the ligand and response strength.

Although, in most cases, ligand binding to LBDs triggers signal transduction within the receptors and leads to physiological responses, an increasing number of reports suggest that some direct-binding ligands act as antagonists without eliciting a response (Silva-Jiménez et al., 2012; Bi et al., 2013; Martín-Mora et al., 2018; Johnson et al., 2021). Due to competitive binding of antagonists with chemoeffectors to chemoreceptor LBDs, discovering direct-binding antagonists of chemoreceptors might provide a useful strategy to inhibit the chemotaxis of pathogenic bacteria.

In this study, to expand the knowledge of chemotactic signal molecules for *C. jejuni*, we first carried out high-throughput screening and microfluidics to identify the novel attractant and antagonist ligands for *C. jejuni* ATCC 33560 Tlp11, and then used the ligand sensing LBD to control the cytosolic part of heterologous proteins, to understand the signal transmission across the membrane upon ligand binding. We performed molecular docking predictions and experimentally verified them to determine the binding between the ligand and chemoreceptor. Finally, based on bioinformatics analysis, we also ascertained the distribution of domains with key residues for ligand recognition in different genera of host-associated commensals or pathogens. To the best of our knowledge, this is the first report on the binding of the dCache-type receptor to methyl pyruvate, toluene, and quinoline. As methyl pyruvate could be detected in humans (Barupal and Fiehn, 2019), the class of chemoreceptors for methyl pyruvate distributed in the order Campylobacterales and their mediated attractant responses might benefit bacterial growth and orient cell bodies to better colonize hosts. The antagonists discovered in this study provide fresh ideas for designing novel inhibitors for inhibiting *C. jejuni* chemotaxis and infection.

## 2 Materials and methods

### 2.1 Strains, plasmids, and growth conditions

The strains and plasmids used in this study are listed in Supplementary Table S1. *C. jejuni* strains were grown in Mueller–Hinton (MH) medium (Duan et al., 2023) and Brucella broth supplemented with 10% filter-sterilized fetal bovine serum (ExCell Bio, China) (Duan et al., 2023) for the growth and chemotaxis experiments, respectively, under microaerobic conditions (85% N_2_, 10% CO_2_, and 5% O_2_), at 37°C. *E. coli* strains were grown in Luria-Bertani medium (Oxoid, United States), at 37°C, for routine culture; in Tryptone broth (1% tryptone and 0.5% NaCl), at 34°C, for the chemotaxis experiments; and in Medium A (Yuan et al., 2017), pH 7.2, at 37°C, for measuring the responses of hybrid kinases in *E. coli* MG1655 strains, under aerobic conditions. The selection medium contained antibiotics, including 10 μg mL^−1^ chloramphenicol and kanamycin for both *C. jejuni* and *E. coli*, 50 μg mL^−1^ ampicillin for *E. coli*, 3 μg mL^−1^ tetracycline for *C. jejuni*, and appropriate concentrations of inducers (Supplementary Table S1).

Plasmids pZQ1-5 and pYFL1-8 were constructed to express the hybrid chemoreceptors and hybrid kinases, respectively. The DNA fragment encoding the LBD of each chimera was amplified from the genomic DNA of *C. jejuni* ATCC 33560. A fragment of the Tar or PhoQ cytoplasmic region was amplified from the genomic DNA of *E. coli* MG1655. Overlap PCR was performed to connect the fragments encoding Tlp11-Tar or Tlp11-PhoQ. The pKG116 plasmid was digested using NdeI and BamHI enzymes and ligated to the amplified fragments Tlp11-Tar or Tlp11-PhoQ by means of Red/ET recombination of *E. coli* GB05-dir (Li et al., 2023). To generate the plasmid pET28b-Tlp11-LBD, the codons of Tlp11-LBD from *C. jejuni* ATCC 33560 were optimized to those preferred by *E. coli* (Ruibiotech, China), and the optimized fragment was ligated to the NdeI- and XhoI-digested pET28b, by means of Red/ET recombination. Point mutations were introduced into the sequence using specific primers and cloned into the corresponding vectors. Plasmids expressing the LBDs from other bacterial species were generated using similar protocols. All plasmids were verified by means of sequencing.

### 2.2 Expression and purification of recombinant proteins

*E. coli* BL21 (DE3) competent cells were transformed with plasmids encoding *C. jejuni* Tlp11-LBD, its mutants, or homologous LBD proteins from other bacterial species. An overnight culture of *E. coli* BL21 (DE3) containing the plasmid was inoculated into 100 mL LB medium containing 10 μg mL^−1^ kanamycin and grown at 37°C, with shaking at 200 rpm. When the OD_600_ reached 0.6 to 0.8, 500 μM isopropyl ß-D-1-thiogalactopyranoside was added into the culture, to induce expression, following which the cells were continually cultured overnight, at 18°C, with shaking at 110 rpm.

For protein purification, *E. coli* cells expressing recombinant proteins were harvested by means of centrifugation at 7,000 rpm, 4°C, for 10 min, to obtain cell pellets, which were then resuspended into Buffer A (25 mM Na_2_HPO_4_, 25 mM NaH_2_PO_4_, and 500 mM NaCl, pH 7.0) and lysed using an ultrahigh-pressure homogenizer (JN-2.5, JNBIO, China). The crushed cells in solution were centrifuged at 20,000 rpm and 4°C for 1.5 h, to remove the insoluble fraction. The soluble supernatant was applied to a 5 mL HisTrap^™^ column (GE Healthcare, United States) equilibrated with Buffer A. The column was washed with different concentrations of Buffer B (25 mM Na_2_HPO_4_, 25 mM NaH_2_PO_4_, 500 mM NaCl, and 500 mM imidazole, pH 7.0) and the eluted proteins were collected using fast protein liquid chromatography (AKTA^™^ Go System, Cytiva, United States). The eluted proteins were validated using sodium dodecyl sulfate-polyacrylamide gel electrophoresis. The target proteins were concentrated using a 10-kDa centrifugal filter (Merck Millipore, United States), and the residual imidazole in the protein solution was removed using a desalting column (GE Healthcare) with Buffer C (25 mM Na_2_HPO_4_, 25 mM NaH_2_PO_4_, and 150 mM NaCl, pH 7.0).

The protein sequences from *Campylobacter coli* (NCBI accession number: WP_201459806.1, residues 32-332), *Helicobacter equorum* (WP_115570384.1, residues 34-332), *Helicobacter himalayensis* (WP_066386874.1, residues 33-320), *Helicobacter mesocricetorum* (WP_199770133.1, residues 31-330), *Helicobacter ganmani* (WP_115552006.1, residues 31-329), and *Campylobacter upsaliensis* (WP_257425542.1, residues 31-331) were purified to obtain Tlp11-LBD homologue proteins.

### 2.3 Thermal shift assay

TSA measurements were performed using a quantitative Real-Time PCR System (LightCycler^®^ 480; Roche, United States), to monitor the *T*_m_ of the LBD proteins. The compounds listed in Supplementary Table S2 were used as ligand candidates for the high-throughput screening. Each 25 μL mixture for the standard assay contained 20 μM protein, 1–2 mM compound in Buffer C, and SYPRO^™^ Orange dye (Life Technologies, United States), at 5× concentration. Samples were heated from 26°C to 85°C, with a ramp rate of 1.2°C min^−1^, to denature the protein. Protein unfolding curves were recorded by detecting the changes in fluorescence. The *T*_m_ values were calculated using first-derivative values (dF/dT) from the raw data.

### 2.4 Microscale thermophoresis

The His-tag dye (MO-L018 RED-tris-NTA, NanoTemper, Germany) was adjusted to a final concentration of 25 nM and used to label the purified protein with a His-tag. The compound was diluted to a series of concentrations in phosphate-buffered saline (Duan et al., 2023) with 0.05% Tween 20 (PBS-T) buffer and mixed in a ratio of 1:1 with protein. The mixtures were loaded into capillaries (Monolith^™^ Series capillaries, NanoTemper) by means of capillary action. Thermophoresis was measured using a Monolith^™^ NT.115 (NanoTemper) instrument, with 40% excitation and 40% MST power configuration. The time-traces of changes in fluorescence, which reflected the thermophoretic movement of labeled protein affected by different concentrations of compound were recorded, and the dose–response curves were fitted with the ‘temperature jump and thermophoresis’ mode. Data analysis was performed using MO. Affinity Analysis version 2.3 (NanoTemper) and Prism version 8.0.2 (GraphPad Software, United States).

### 2.5 Construction of *Campylobacter jejuni* WT NCTC 11168ΩTlp11 and ΔCheY strains

The *C. jejuni* WT NCTC 11168 was used to generate the 11168ΩTlp11 strain by means of double-crossover homologous recombination, using a suicide plasmid containing homologous arms that flanked the target gene (Duan et al., 2023). The insertion site for *tlp11* was selected between 16 s RNA and 23 s RNA in the genome of *C. jejuni* NCTC 11168. The linear fragment *16sRNAup-tlp11-km-23sRNAdown*, which contained 1,044 bp of the upstream *16sRNA*, full-length *tlp11* with its promoter, a kanamycin resistance gene, and 1,026 bp of the downstream *23sRNA*, was constructed using overlap PCR. The fragment *16sRNAup-tlp11-km-23sRNAdown* was transferred into *E. coli* GB08-red containing pBJ114, to generate the plasmid pBJ110-Ω*tlp11*. The inserted sequences in the transformants were verified by means of enzyme digestion and sequencing. pBJ110-Ω*tlp11* was electroporated into *C. jejuni* NCTC 11168 using a modified protocol adapted from a previous report (Duan et al., 2023), and screened using kanamycin, to obtain *C. jejuni* NCTC 11168ΩTlp11. To construct the 11168ΩTlp11/ΔCheY strain, the *cheYup-cm*-*cheYdown* fragment containing 1 kb homologous arms of upstream and downstream *cheY* and the chloramphenicol resistance gene, was ligated into pBJ114 by means of Red/ET recombination, to generate the plasmid pBJ110-Δ*cheY*. Next, pBJ110-Δ*cheY* was electroporated into *C. jejuni* NCTC 11168ΩTlp11 competent cells and screened with chloramphenicol, to generate *C. jejuni* NCTC 11168ΩTlp11/ΔCheY. The *C. jejuni* mutants were verified by means of PCR and DNA sequencing.

### 2.6 The effect of methyl pyruvate and pyruvate on the growth of *Campylobacter jejuni*

The *C. jejuni* ATCC 33560, *C. jejuni* WT NCTC 11168 and 11168ΩTlp11 cells were grown on a MH agar plate overnight, at 37°C, under microaerobic conditions. The colonies were suspended in PBS (Duan et al., 2023), pH 7.2, inoculated into Minimal Essential Medium broth (Life Technologies) containing 10% fetal bovine serum to an initial OD_600_ of 0.01, and grown at 37°C, 100 rpm, under microaerobic conditions. When required, Minimal Essential Medium was supplemented with different concentrations of methyl pyruvate or pyruvate (both from Macklin, China).

### 2.7 Measurements of the spreading of *Campylobacter jejuni* strains

The *C. jejuni* WT NCTC 11168, 11168ΩTlp11, and 11168ΔCheY cells were cultured on MH agar plates, at 37°C, for 18–24 h, under microaerobic conditions. The colonies were then harvested and resuspended in MH broth, until an OD_600_ of 0.05 was attained. Semi-solid MH agar (0.4%) was used to observe the spreading of *C. jejuni*. Briefly, 1 μL of the cell solutions were added to the semi-solid MH agar plates and incubated at 37°C, for 48 h, under microaerobic conditions. The diameter of the colony ring on the semi-solid MH agar plate was measured.

### 2.8 Microfluidic experiments

The *E. coli* cells expressing hybrid chemoreceptor and GFP were grown in tryptone broth, up to an OD_600_ of 0.55, at 34°C, with shaking at 250 rpm. The cells were harvested by means of centrifugation at 5,000 rpm, washed twice with Tethering Buffer (10 mM KH_2_PO_4_, 10 mM K_2_HPO_4_, and 0.1 mM EDTA, pH 7.0), and resuspended in Tethering Buffer such that an OD_600_ of 5.5 was attained. The chemotactic responses of *E. coli* cells to the compounds were measured using a microfluidic device (Si et al., 2012). The collected *E. coli* cells were added to the sink pores of the device and allowed to freely diffuse into the observation channel for 40 min. Subsequently, the compound solution was added to the source pores, to establish a concentration gradient gradually in the observation channel of the device. Fluorescence intensity in the observation channel was detected using an LSM 800 laser scanning confocal microscope (Zeiss, Germany). Data were analyzed using ImageJ software.

*C. jejuni* chemotaxis assays were performed using the microfluidic device described above. *C. jejuni* was grown in Brucella broth containing 10% fetal bovine serum, at 37°C, with shaking at 100 rpm, under microaerobic conditions, until an OD_600_ of 0.2 was attained. The cells were collected by means of centrifugation at 3,000 rpm for 5 min, and resuspended in PBS, such that an OD_600_ of 2.0 was attained. The collected *C. jejuni* cells were loaded into sink pores and allowed to diffuse into the observation channel. After 20 min, compound solutions were added to the source pores, to form a concentration gradient. Chemotactic responses were observed in the phase-contrast mode, using an inverted fluorescence microscope (TI-E, Nikon, Japan). The results were analyzed by counting the numbers of bacteria, about 200–600 cells, in the analysis region (100 × 100 μm) of the observation channel of microfluidic device. Data analysis was performed using Prism version 8.0.2.

### 2.9 Construction of hybrid kinases in engineered two-component systems

The promoters of *mgtLA* and *ompC* were amplified from the *E. coli* MG1655 genome and attached to the *gfp* gene by means of overlap PCR, to obtain P*_mgtLA_-gfp* and P*_ompC_-gfp*. The linear fragment, P*_mgtLA_-gfp* or P*_ompC_-gfp*, was cloned into the vector pUA66 at the XhoI and BamHI restriction sites, to generate pUA66-P*_mgtLA_*-GFP and pUA66-P*_ompC_*-GFP, respectively, as plasmids encoding the reporter. These reporter plasmids were then transformed into *E. coli* MG1655, and the fluorescence intensity triggered by 400 mM NaCl (mediated via the PhoQ-PhoP-GFP system) or 20% sucrose (mediated via the EnvZ-OmpR-GFP system) was detected using an Imaging Flow Cytometer (ImageStream^X^ Mark II, Merck, United States).

The reporter plasmid pUA66-P*_mgtLA_*-GFP and the plasmid encoding the hybrid kinase were together transformed into *E. coli* MG1655/ΔPhoQ strain. *E. coli* cells containing the reporter and Tlp11-PhoQ chimera were incubated in Medium A, at 37°C, with shaking (200 rpm), until an OD_600_ of 0.4 was attained, following which different concentrations of methyl pyruvate were added to the medium and the cells were incubated for 40 min. The fluorescence intensity of the cells was monitored using a plate reader (Varioskan^™^ LUX, Life Technologies) equipped with a 488 nm laser and a 528/12 bandpass filter. The acquired data were analyzed using Prism 8.0.2.

### 2.10 Construction of the *Escherichia coli* MG1655/ΔphoQ strain

The linear fragment *phoQup-tet-phoQdown*, which contained 500 bp of the upstream *phoQ*, a tetracycline resistance gene, and 500 bp of the downstream *phoQ*, was generated by means of overlap PCR, and then ligated into pRE112 by means of Red/ET recombination, to construct the plasmid pRE112-Δ*phoQ*. The helper plasmid pTKRED (Lange et al., 2019) was transformed into *E. coli* MG1655, rendering the cells resistant to spectinomycin. Transformed cells were cultured in LB with 60 μg mL^−1^ spectinomycin, culture at 30°C, 200 rpm, to an OD_600_ of 0.2, then add 0.5 mM IPTG to an OD_600_ of 0.5. The cells were harvested to produce competent cells, and the linear fragment *phoQup-tet-phoQdown* amplified from pRE112-Δ*phoQ* was transformed by electroporation into the *E. coli* MG1655-pTKRED competent cells and screened on agar plates containing tetracycline and spectinomycin. The pTKRED plasmid was removed after 3 generations at 42°C on agar plates containing tetracycline. The mutant cells obtained were validated using PCR and sequencing molecular dynamics.

### 2.11 Molecular dynamics simulations

The three-dimensional structure of Tlp11-LBD was predicted using AlphaFold 2 (Jumper et al., 2021). MD simulations were conducted for conformation optimization of Tlp11-LBD, using GROMACS 2021.5 package (Sarkar et al., 2022). Tlp11-LBD was parameterized using the Amberff14sb force field (Maier et al., 2015). A cubic box was established by extending at least 1.4 nm outward along the protein (10 × 10 × 10 nm^3^), following which the system was solvated in TIP3P water, and 0.15 M NaCl (99 Na^+^ and 90 Cl^−^) was added to maintain electrical neutrality. Energy minimization was performed using the steepest descent algorithm, with a force tolerance of 500 kJ mol^−1^ nm^−1^. Periodic boundary conditions were imposed in all the three directions. The system was relaxed for 1 ns under the NPT ensemble and position restraints with a constant of 1,000 kJ mol^−1^ nm^−2^ in three directions were applied to the heavy atoms of the protein.

After completing the above steps, 100 ns NPT MD simulations were performed. The pressure was maintained at 1 bar using a Parrinello-Rahman barostat (Braga and Travis, 2006), in an isotropic manner, while the temperature was maintained at 310 K using a V-rescal thermostat (Bussi et al., 2007). The LINCS algorithm was used to constrain the bond lengths of hydrogen atoms (Hess, 2008). Lennard-Jones interactions were calculated within a cutoff of 1.2 nm, and electrostatic interactions beyond 1.2 nm were treated using the particle-mesh Ewald method, with a grid spacing of 0.16 nm. PyMOL (Seeliger and De Groot, 2010) was used to visualize the results.

### 2.12 Molecular docking

Molecular docking was performed using AutoDockTools-1.5.6 (Sousa et al., 2006), to predict the interaction between Tlp11-LBD and compounds. A docking box was constructed around the membrane-distal and -proximal pockets of Tlp11-LBD, as docking sites for compounds. The optimal configuration was determined by calculating the binding free energies of each conformation. The docking results were displayed using PyMOL.

### 2.13 Circular dichroism spectroscopy

Tlp11-LBD and its mutants were dissolved in Buffer D (25 mM Na_2_HPO_4_, 25 mM NaH_2_PO_4_, pH 7.0). Far-ultraviolet CD spectra were recorded with the protein concentration of 0.06 mg mL^−1^, at 25°C, with a scan rate of 100 nm min^−1^, in the wavelength range of 190–240 nm, using a JASCO J-1500 CD spectrometer (JASCO, Japan). The spectra were corrected using a solvent. The curves were smoothed using Prism version 8.0.2.

### 2.14 Bioinformatics analysis

The Tlp11-LBD homologues were searched against the NCBI RefSeq Database (Tatusova et al., 2014). The Tlp11-LBD (NCBI accession number: AZU51669.1) sequence from *C. jejuni* ATCC 33560 was used as a query in a BLAST search against the NCBI RefSeq Database, and the maximum target sequence was set to 5,000, using default parameters. Tlp11-LBD homologue sequences were aligned using ClustalOmega.^1^ A phylogenetic tree was constructed using the neighbor-joining method in MEGA 7.0.26 (Filipski et al., 2014), with 1,000 bootstraps, and the results were displayed in iTOL version 6 (Letunic and Bork, 2021). Conservation pattern analysis of Tlp11-LBD homologues was conducted using WebLogo 3 server.^2^ Amino acids were numbered based on the *C. jejuni* ATCC 33560 Tlp11 sequence. The results of the multiple sequence alignments have been displayed using GeneDoc (Lanave et al., 2002).

## 3 Results

### 3.1 High-throughput screening of the ligand for *Campylobacter jejuni* chemoreceptor Tlp11

**Figure 1:**
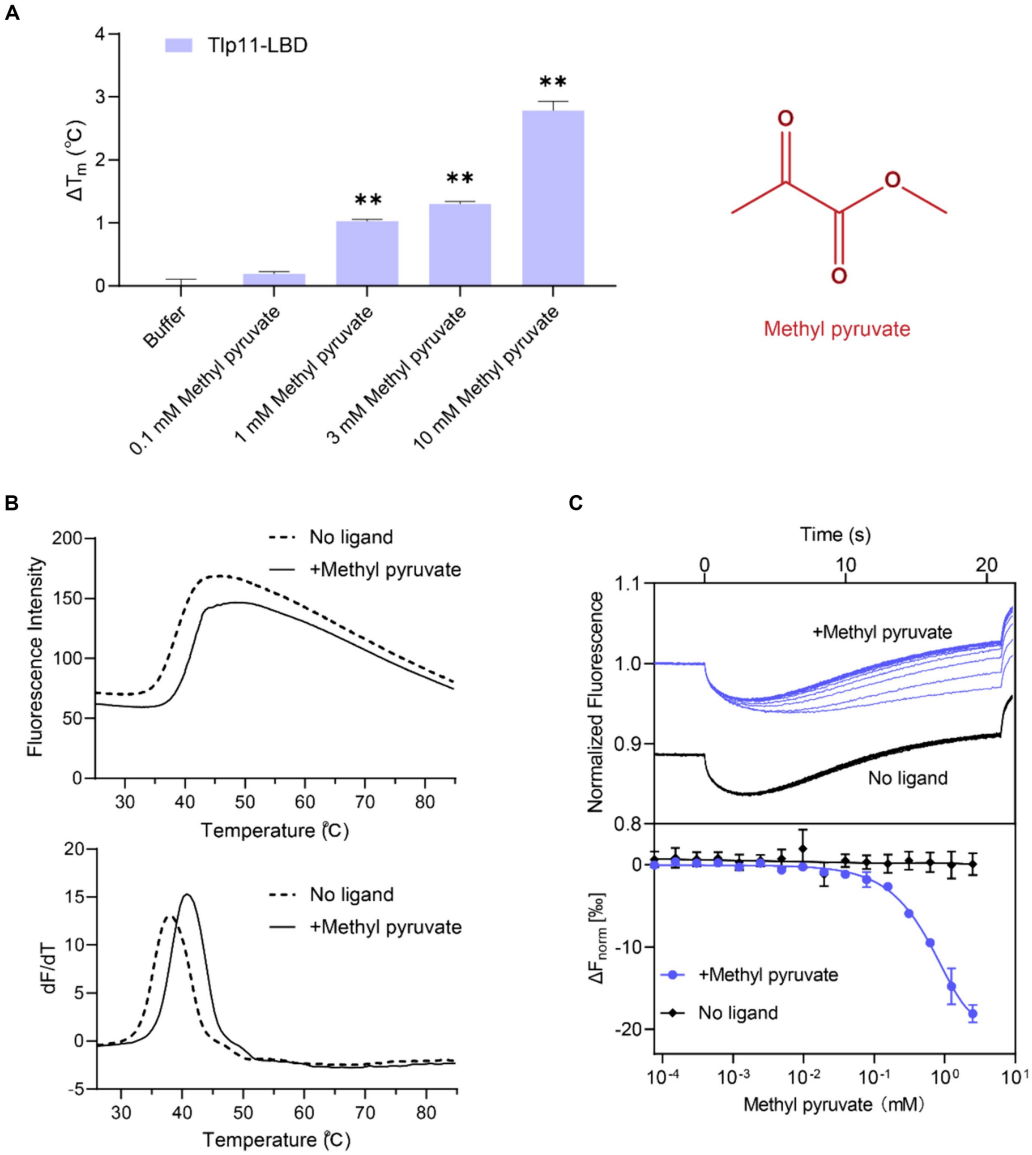
Identification of methyl pyruvate as the direct-binding ligand of Tlp11-LBD. **(A)** The effect of different concentrations of methyl pyruvate on the *T*_m_ of Tlp11-LBD. Each concentration of methyl pyruvate indicated in **(A)** was the working concentration in the system. The right panel shows the molecular structure of methyl pyruvate. Error bars represent the standard errors of three independent replicates, shown as mean ± SD. The *p*-values were calculated using the paired *t*-test; **p < 0.01, compared to buffer. **(B)** The thermal unfolding curves and calculated Δ*T*_m_ of thermal shift assay measurements for Tlp11-LBD, in the absence and presence of 10 mM methyl pyruvate. **(C)** Microscale thermophoresis of Tlp11-LBD with methyl pyruvate. The blue and black lines indicate the thermophoresis of Tlp11-LBD labeled with fluorescent dyes, at different concentrations of methyl pyruvate and in the buffer, respectively. The working concentration of the proteins used for MST detection was 250 nM. The maximum working concentration of the ligand was 2.5 mM, and it was gradually diluted. Upper panel: raw thermophoretic data; lower panel: dose-response curves with fitting results. Error bars represent the standard errors of three independent replicates, shown as mean ± SD.

Fluorescence-based TSA, which has been widely used to discover direct-binding molecules of proteins with high efficiency (Fernández et al., 2018), was used to identify the ligand specificity of the *C. jejuni* chemoreceptor Tlp11. The Tlp11-LBD protein (residues 32–332 of Tlp11) from the strain *C. jejuni* ATCC 33560 was expressed and purified from *E. coli*. Hydrophobic reactive dyes were added to the purified protein sample, and the protein was heated to record the change in fluorescence intensity as the temperature increased during the denaturation process of the protein (see Materials and Methods). The melting temperature (*T*_m_) of the target protein was obtained and represented as the midpoint of the protein unfolding transition. Most ligands stabilize the protein during binding, leading to an increase in *T*_m_. Using TSA, we screened a compound library composed of 131 molecules (Supplementary Table S2) that commonly act as microbial carbon and nitrogen sources or metabolites in the human GI tract. Among the compounds in the library, methyl pyruvate elicited the most significant increase in *T*_m_. In the absence of the ligand, the *T*_m_ of Tlp11-LBD was 38.3°C, while in the presence of 1 mM methyl pyruvate, the *T*_m_ increased by 1.1°C compared to buffer (Figure 1A). The elevated *T*_m_ of Tlp11-LBD was concentration-dependent, with an increased concentration of methyl pyruvate triggering a larger Δ*T*_m_ (Δ*T*_m_ of 1.3°C and 2.8°C at 3 mM and 10 mM methyl pyruvate, respectively; Figures 1A,B), indicating that methyl pyruvate might bind to Tlp11-LBD as a ligand.

### 3.2 Measurement of the binding affinity of methyl pyruvate towards Tlp11-LBD

We measured the *in vitro* binding affinity of methyl pyruvate for *C. jejuni* ATCC 33560 Tlp11-LBD protein using MST. MST quantifies interaction affinities by detecting the direct movement of fluorescent molecules along temperature gradients in capillaries. Ligand binding changes the thermophoretic movement of proteins, which can be used to derive *Kds*, by sequentially scanning capillaries with different ligand concentrations. MST has been applied to the study of weak binding systems and validated to provide reliable results (Linke et al., 2016; Gao et al., 2021). We observed that compared to the negative control in the buffer, supplementation with different concentrations of methyl pyruvate significantly affected the thermophoresis of Tlp11-LBD in the MST experiments (Figure 1C). The derived *Kd* of Tlp11-LBD binding to methyl pyruvate was 688 ± 140 μM at pH 7.0, indicating that methyl pyruvate is a direct-binding ligand for Tlp11-LBD. The *Kd* of methyl pyruvate measured by MST was consistent with the rapid increase in Δ*T*_m_ of Tlp11-LBD elicited by 100 μM to 1 mM methyl pyruvate observed in the TSA experiments (Figure 1A). We also tested the binding abilities of methyl pyruvate analogues to Tlp11-LBD using MST (Supplementary Table S3). Except for ethyl pyruvate that could bind to Tlp11-LBD with a *Kd* of 2.36 ± 0.17 mM, the other analogues, including pyruvate, did not bind to Tlp11-LBD (Supplementary Figures S1A–G), suggesting that Tlp11-LBD has a high specificity for binding to methyl pyruvate.

### 3.3 *Campylobacter jejuni* Tlp11 mediates a chemoattractant response towards methyl pyruvate

**Figure 2:**
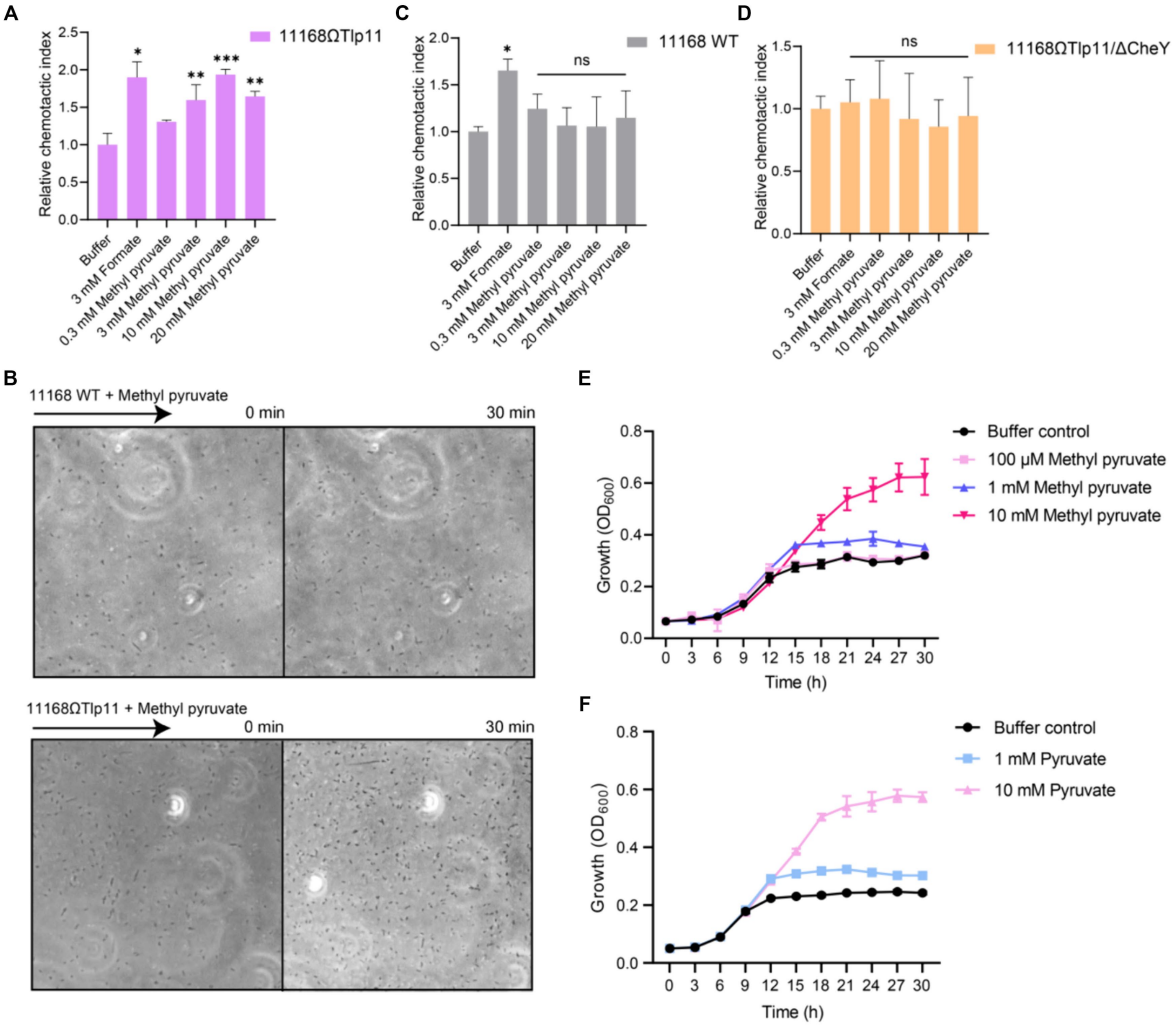
The effect of methyl pyruvate on chemotaxis and growth of *C. jejuni*. **(A,C,D)** The chemotactic responses of the *C. jejuni* NCTC 11168 strain expressing Tlp11 of ATCC 33560 (11168ΩTlp11) **(A)**, WT NCTC 11168 strain **(C)**, and non-chemotactic mutant 11168ΩTlp11/ΔCheY strain **(D)** towards different concentrations of methyl pyruvate, shown as relative chemotactic index. Error bars represent the standard errors of three independent replicates, shown as mean ± SD. The *p*-values were calculated using the paired *t*-test; *p < 0.05, **p < 0.01, and ***p < 0.001, compared to buffer. **(B)** Examples of the distributions of the *C. jejuni* NCTC 11168 WT or ΩTlp11 cells in the observation channel of the microfluidic device, obtained before addition of ligand and 30 min after response to 10 mM methyl pyruvate. The black arrow indicates the direction up the concentration gradient of methyl pyruvate. The response is characterized by measurement of the cell number (~200–600 cells) in the analysis region of the observation channel, as the view in **(B)**. **(E,F)** The growth curves of the *C. jejuni* ATCC 33560 strain in Minimal Essential Medium supplemented with fetal bovine serum and different concentrations of methyl pyruvate **(E)** and pyruvate **(F)**. Error bars represent the standard errors of 4 independent replicates, shown as mean ± SD.

To understand the correlation between the physiological function and specific interaction of Tlp11 with methyl pyruvate, we detected the chemotactic responses of *C. jejuni* towards methyl pyruvate. Although *C. jejuni* ATCC 33560 encodes Tlp11 and has motile and invasive abilities (Konkel and Joens, 1990; Hazeleger et al., 1998), difficulties in the genetic operation of this strain make it difficult to achieve functional verification of Tlp11. Moreover, ATCC 33560 is more sensitive to oxygen, which makes it difficult to perform chemotaxis measurements using this strain in our microfluidic assays. Therefore, we expressed Tlp11 of ATCC 33560 in *C. jejuni* NCTC 11168, which does not encode Tlp11, but has been widely used to study *C. jejuni* chemotaxis in recent studies (Hartley-Tassell et al., 2010; Duan et al., 2023), to obtain the Tlp11-expressing strain 11168ΩTlp11. Although the spreading ring of 11168ΩTlp11 cells on the semi-solid MH agar plate was smaller than that of WT NCTC 11168 (Supplementary Figure S2), 11168ΩTlp11 and 11,168 WT had a similar chemotactic strength to formate (Figures 2A,C), indicating that the overexpression of Tlp11 in NCTC 11168 might induce a higher frequency of tumbling. 11,168 ΔCheY strain, which was reported with low motile ability (Chandrashekhar et al., 2015), had a much smaller spreading ring than 11168ΩTlp11 (Supplementary Figure S2).

We measured the responses of 11168ΩTlp11 cells towards methyl pyruvate using a microfluidic device reported previously (Supplementary Figure S3) (Duan et al., 2023). *C. jejuni* cells were loaded into the sink pores of the device and allowed to swim into the observation channel. The methyl pyruvate solution was loaded into the source pores and allowed to diffuse through the agarose gel into the observation channel, to form a concentration gradient. If methyl pyruvate is the attractant, *C. jejuni* cells sense the attractant gradient and move from the sink pore into the observation channel, thereby accumulating towards the source. However, if it is the repellent, cells move out of the observation channel towards the sink pore, thereby decreasing the cell intensity in the observation channel. The cell number was counted (~200–600 cells) in each of the analysis region (100 × 100 μm) of the observation channel in microfluidic device, in response to the ligand or without ligand (buffer), for 30 min. The number of cells in response to ligand gradient was normalized to that before adding the ligand, to obtain the chemotactic index (CI). The CI of cells in response to ligand gradient was then normalized to that of cells in the buffer, to get relative CI. A relative CI >1 indicates an attractant response, whereas a relative CI <1 indicates a repellent response.

We used formate, a reported attractant for the *C. jejuni* chemoreceptor Tlp1, as the positive control (Vegge et al., 2009; Duan et al., 2023), and a blank buffer as the negative control, for measuring the chemotactic response. The 11168ΩTlp11 cells swam up the formate gradient in the observation channel, and the number of accumulated cells increased over time, indicating an attractant response to formate (Figure 2A). In contrast, cell density remained almost unchanged in the blank buffer. Similar to the response towards formate, 11168ΩTlp11 cells exhibited a robust attractant response towards methyl pyruvate, with cells moving up the gradient and accumulating in the observation channel (Figures 2A,B). This attractant response was concentration-dependent, indicating that methyl pyruvate is a novel attractant for ΩTlp11. In contrast, although the WT NCTC 11168 cells also exhibited an attractive response to formate, they failed to show any chemotaxis towards methyl pyruvate (Figures 2B,C), suggesting that the WT NCTC 11168 cells could not respond to methyl pyruvate, and the attractant response of ΩTlp11 towards methyl pyruvate is mediated specifically via Tlp11.

As a control, the non-chemotactic mutant of 11168ΩTlp11 with CheY deletion (11168ΩTlp11/ΔCheY) showed no chemotaxis towards formate or methyl pyruvate (Figure 2D), indicating that the chemotaxis system mediates the response towards methyl pyruvate in 11168ΩTlp11. Therefore, our results demonstrated that the chemoreceptor Tlp11 of *C. jejuni* ATCC 33560 mediates the attractant response towards methyl pyruvate through a chemotaxis system.

### 3.4 Methyl pyruvate promotes the growth of *Campylobacter jejuni*

To understand the physiological significance of the attractant response to methyl pyruvate, we explored the effects of methyl pyruvate on the growth of *C. jejuni*. A previous study suggested that methyl pyruvate improves *C. jejuni* growth (Wagley et al., 2014). We measured the growth curves of the *C. jejuni* ATCC 33560 strain in Minimal Essential Medium supplemented with different concentrations of methyl pyruvate or pyruvate under the shaking condition. The results showed that at concentrations above 1 mM, methyl pyruvate significantly promoted the growth of *C. jejuni* ATCC 33560 (Figure 2E). This is possibly because methyl pyruvate can be converted to pyruvate, the product of glycolysis, and metabolized by the tricarboxylic acid cycle, to serve as a carbon source for *C. jejuni* growth, as suggested by a previous study (Wagley et al., 2014). Similar to that observed for methyl pyruvate, pyruvate-mediated promotion of the growth was also observed (Figure 2F). Therefore, methyl pyruvate has metabolic value, and its chemotaxis may be beneficial for the growth of *C. jejuni*. In addition, the WT NCTC 11168 and 11168ΩTlp11 showed similar growth in addition of methyl pyruvate (Supplementary Figure S4), indicating that the presence of Tlp11 does not affect the *C. jejuni* methyl pyruvate growth response under the shaking condition.

### 3.5 Sensing of methyl pyruvate by Tlp11-LBD elicits signal transduction in the Tlp11-tar hybrid chemoreceptor

**Figure 3:**
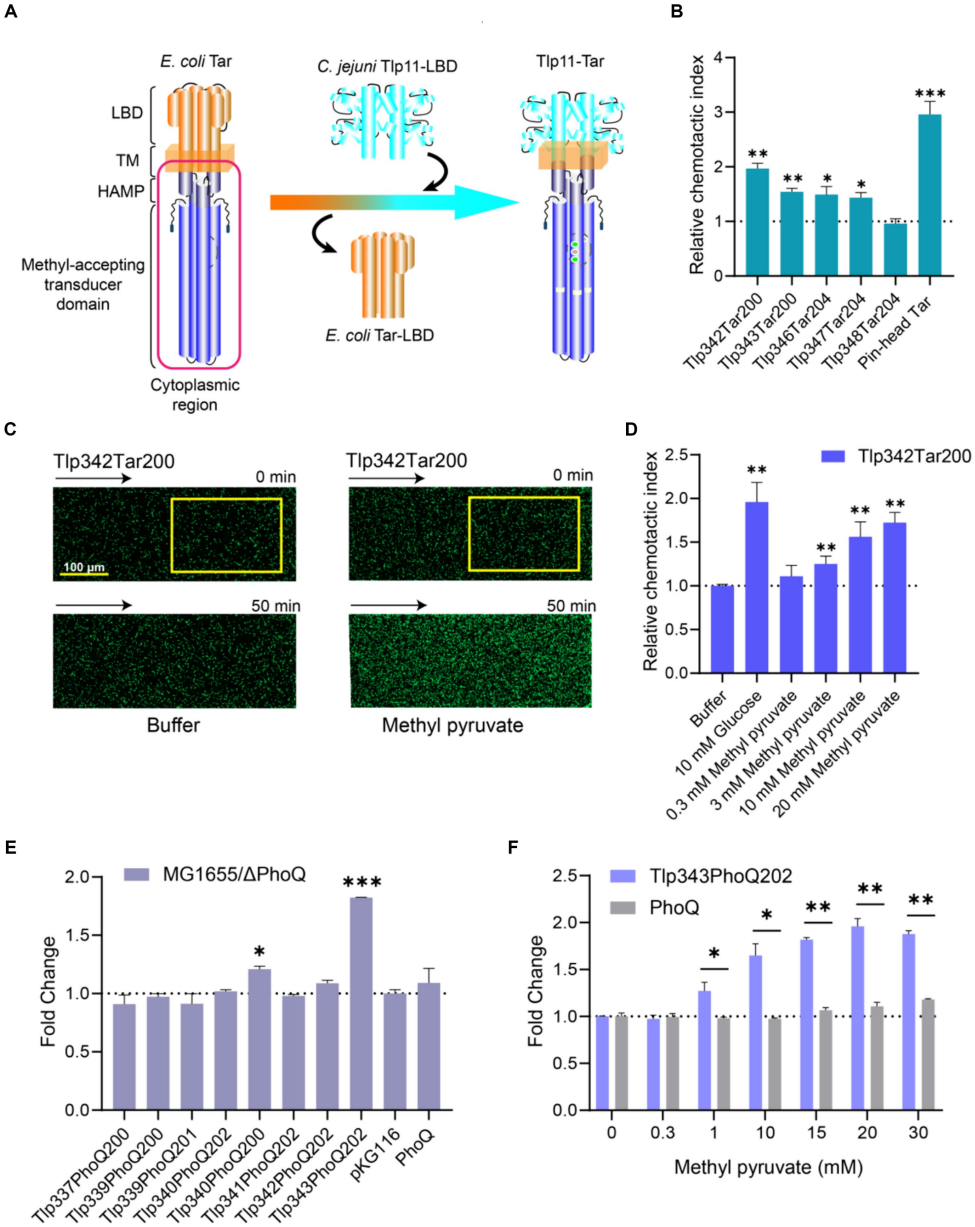
Response of chimeras Tlp11-Tar and Tlp11-PhoQ to methyl pyruvate. **(A)** Design and construction of the Tlp11-Tar hybrid receptor. On the left is the schematic diagram of the *E. coli* chemoreceptor Tar, with the cytoplasmic region shown in red. *C. jejuni* Tlp11-LBD is connected to the cytoplasmic region of Tar to form the hybrid receptor Tlp11-Tar. The fusion site is located in the TM2. **(B)** The relative chemotactic index (CI) of the *E. coli* VS188 cells expressing Tlp342Tar200, Tlp343Tar200, Tlp346Tar204, Tlp347Tar204, Tlp348Tar204, or pin-head Tar as the sole receptor, in response to 10 mM glucose for 50 min. **(C)** Examples of the distribution of the *E. coli* cells expressing Tlp342Tar200 in the observation channel of the microfluidic device, acquired before addition of the ligand and 50 min after the response to 20 mM methyl pyruvate (scale bar: 100 μm). The *x*-component (black arrow) indicates the direction up the concentration gradient of methyl pyruvate. The response is characterized by measurements of the total fluorescence intensity (cell density) in the analysis region (225 × 150 μm) of the observation channel, indicated by a yellow rectangle. **(D)** The relative CI of *E. coli* VS188 cells expressing Tlp342Tar200 as the sole receptor, in response to the indicated concentrations of methyl pyruvate or buffer at 50 min. In **(B,D)**, the corresponding values of the fluorescence intensities in the analysis regions were normalized to the fluorescence intensity of cells before adding the compound, to obtain CI. The CI of cells in response to compound gradient was then normalized to that of cells in the buffer, to get the relative CI. **(E)** The fold-change in fluorescence intensity of *E. coli* MG1655/ΔPhoQ expressing each hybrid kinase, PhoQ, or only containing empty vector pKG116, after stimulation with 20 mM methyl pyruvate for 40 min. **(F)** The responses of *E. coli* MG1655/ΔPhoQ expressing Tlp343PhoQ202 as the single chimera or PhoQ towards indicated concentrations of methyl pyruvate. Error bars represent the standard errors of three independent replicates, shown as mean ± SD. The *p*-values were calculated using the paired *t*-test; *p < 0.05, **p < 0.01, and ***p < 0.001.

To determine whether methyl pyruvate stimulates transmembrane signaling through Tlp11-LBD, we fused *C. jejuni* ATCC 33560 Tlp11-LBD with the cytoplasmic region of the *E. coli* chemoreceptor Tar to construct Tlp11-Tar hybrid chemoreceptors (Figure 3A). Five hybrid receptors with different fusion positions in the second transmembrane helix (TM2) were obtained: Tlp11[1-342]-Tar[200-553] (Tlp342Tar200), Tlp11[1-343]-Tar[200-553] (Tlp343Tar200), Tlp11[1-346]-Tar[204-553] (Tlp346Tar204), Tlp11[1-347]-Tar[204-553] (Tlp347Tar204), and Tlp11[1-348]-Tar[204-553] (Tlp348Tar204) (Supplementary Figure S5A). To verify the activities of these hybrid chemoreceptors, we expressed each protein as a chimera in green fluorescent protein (GFP)-labeled *E. coli* VS188 without any other chemoreceptors, such that each hybrid receptor served as the only chemoreceptor in the *E. coli*. Glucose was used as the effector to screen for hybrid receptor activity, as it is a substrate of the phosphotransferase system that stimulates functional chemoreceptors in the receptor-CheA-CheW ternary complex and triggers an attractant response (Somavanshi et al., 2016; Bi and Sourjik, 2018). These chemotactic responses triggered by glucose through the phosphotransferase system are independent of the chemoreceptor LBD (Neumann et al., 2012). Using the microfluidic device described above, we observed that of the five hybrid receptors, GFP-labeled *E. coli* expressing Tlp342Tar200 showed the strongest chemotaxis in the glucose gradient (Figure 3B), suggesting that Tlp342Tar200 had the best communication with the *E. coli* chemosensory pathway. Thus, we used this hybrid receptor for subsequent chemotaxis measurements.

Observation of the response of *E. coli* expressing Tlp342Tar200 as the sole receptor for methyl pyruvate revealed that Tlp342Tar200 mediated a strong attractant response to methyl pyruvate, with the cells moving up the methyl pyruvate gradient and accumulating in the observation channel (Figure 3C), in a concentration-dependent manner (Figure 3D). As a control, cells expressing the pin-head Tar (Tar without LBD) (Bi et al., 2016) or WT Tar as the sole chemoreceptor showed no response or a much weaker response to methyl pyruvate (Supplementary Figures S5B,C), thus supporting that methyl pyruvate is a signaling molecule specifically sensed by Tlp11-LBD, and its binding can stimulate transmembrane signaling in Tlp11.

### 3.6 Methyl pyruvate sensed by Tlp11-LBD triggers signal transduction in an engineered TCS

To further explore the signal transduction ability of Tlp11-LBD in sensing methyl pyruvate, we enrolled *C. jejuni* ATCC 33560 Tlp11-LBD in engineered TCSs, to evaluate its response to methyl pyruvate, using the fluorescence intensity of GFP as a reporter. *E. coli* PhoQ/PhoP and EnvZ/OmpR were chosen as the target TCSs for the first design. To construct the signal output module, *gfp* was placed under the control of the promoter region of *mgtLA*, which is regulated by PhoP, or the promoter of *ompC*, which is regulated by OmpR (Supplementary Figure S6). *E. coli* MG1655 expressing the PhoQ-PhoP-GFP system showed a clear response to osmotic pressure triggered by 400 mM NaCl (Supplementary Figure S7A), the stimulus reported for PhoQ (Yuan et al., 2017). However, the EnvZ-OmpR-GFP system showed fluorescence leakage and could not respond properly to the osmotic pressure triggered by 20% sucrose (Wang et al., 2012) (Supplementary Figure S7B); therefore, we utilized the PhoQ-PhoP-GFP system for the subsequent design.

To construct the signal input module, we fused the Tlp11-LBD with the PhoQ cytoplasmic region, to design the Tlp11-PhoQ hybrid kinases (Supplementary Figure S6), Tlp11[1-337]-PhoQ[200-486] (Tlp337PhoQ200), Tlp11[1-339]-PhoQ[200-486] (Tlp339PhoQ200), Tlp11[1-339]-PhoQ[201-486] (Tlp339PhoQ201), Tlp11[1-340]-PhoQ[202-486] (Tlp340PhoQ202), Tlp11[1-340]-PhoQ[200-486] (Tlp340PhoQ200), Tlp11[1-341]-PhoQ[202-486] (Tlp341PhoQ202), Tlp11[1-342]-PhoQ[202-86] (Tlp342PhoQ202), Tlp11[1-343]-PhoQ[202-486] (Tlp343PhoQ202), which connected Tlp11 and PhoQ in TM2 (Supplementary Figure S7C). The activity of each chimera in sensing methyl pyruvate was determined by measuring the fluorescence intensity of GFP in the *E. coli* MG1655/ΔPhoQ strain. Cells expressing Tlp343PhoQ202 showed the largest increase in fluorescence intensity upon addition of methyl pyruvate, with the highest fold-change representing the ratio of fluorescence intensity in the presence of methyl pyruvate to that without methyl pyruvate, normalized to cell density (Figure 3E). Moreover, the response of Tlp343PhoQ202 towards methyl pyruvate exhibited significant concentration-dependence (Figure 3F), suggesting that Tlp343PhoQ202 senses methyl pyruvate and triggers downstream signals. However, control cells expressing full-length PhoQ or an empty vector showed almost no response towards methyl pyruvate (Figures 3E,F). These results indicated that the signal transduction elicited by methyl pyruvate occurs through Tlp11-LBD.

### 3.7 Computational prediction and validation of the binding mode of methyl pyruvate towards Tlp11

**Figure 4:**
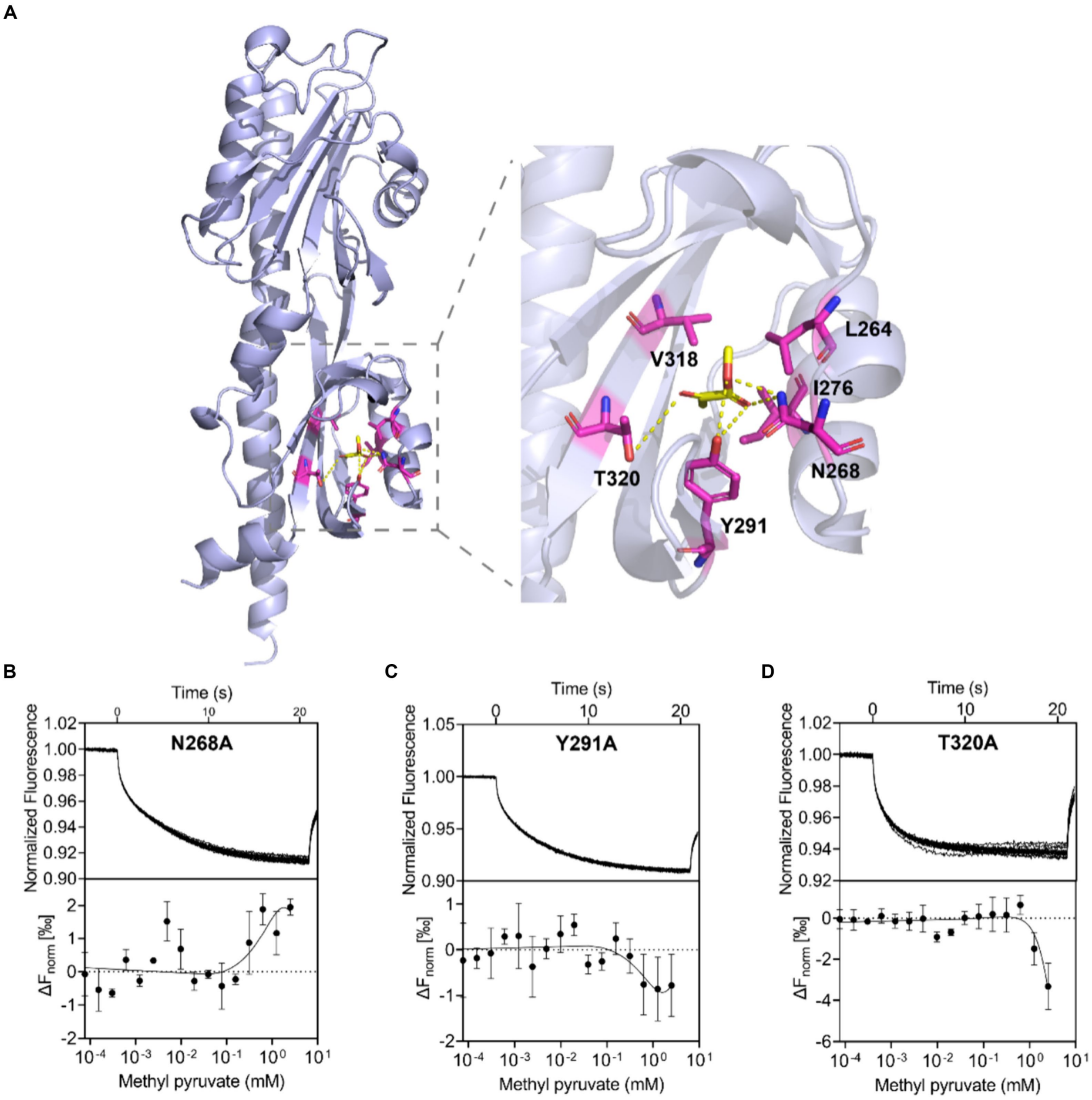
Binding interaction analysis using molecular docking and Tlp11-LBD mutant proteins. **(A)** Molecular docking analysis of the interaction of Tlp11-LBD with methyl pyruvate using Autodock. The conformation with the lowest binding free energy is shown with PyMOL. Methyl pyruvate is predicted to bind to the membrane-proximal pocket of Tlp11-LBD. The key residues in the ligand-binding pocket involved in methyl pyruvate binding are shown as sticks. The hydrogen bonds are shown as yellow dashed lines. **(B–D)** MST measurements for the interactions of Tlp11-LBD mutants N268A, Y291A, and T320A with methyl pyruvate. The upper panel indicates the representative curves for thermophoresis of mutant proteins with different concentrations of methyl pyruvate, while the lower panel indicates the dose–response curve with the fitting result. Error bars represent the standard errors of three independent replicates, shown as mean ± SD. The concentration for the mutant proteins was 250 nM, and the maximum concentration for the ligand was 2.5 mM, which was diluted gradually.

To understand how methyl pyruvate binds to *C. jejuni* ATCC 33560 Tlp11-LBD, the structure of Tlp11-LBD was modeled using Alphafold 2 (Jumper et al., 2021) and molecular dynamics (MD) simulations were conducted to optimize the conformation obtained (Supplementary Figure S8). The binding free energy for each predicted binding mode of methyl pyruvate to Tlp11-LBD was calculated by means of molecular docking. We tried to dock methyl pyruvate into the pockets of both membrane-proximal and -distal subdomains of Tlp11-LBD, and found that it could only be docked into the membrane-proximal pocket, and the lowest binding free energy was −3.04 kcal mol^−1^. In addition, the predicted lowest binding free energy of pyruvate interacting with Tlp11-LBD membrane-proximal pocket was −1.39 kcal mol^−1^, consistent with the MST result that the binding of pyruvate to Tlp11-LBD was not detectable (Supplementary Figure S1C). The docking results showed that methyl pyruvate may interact with the residues L264, N268, I276, Y291, V318, and T320 in the membrane-proximal pocket, at a distance of <3.5 Å. The ester and keto groups of methyl pyruvate formed hydrogen bonds with the residues N268, Y291, and T320. The residues L264, I276, and V318 were also spatially adjacent, and may have interacted with methyl pyruvate through hydrophobic interactions (Figure 4A).

In order to confirm the contribution of L264, N268, I276, Y291, V318, and T320 to the binding to methyl pyruvate, we generated Tlp11-LBD proteins with an alanine point mutation at individual residues, to obtain the mutant proteins L264A, N268A, I276A, Y291A, V318A, and T320A, and then analyzed the binding affinities of methyl pyruvate to these mutants using MST. These mutant proteins significantly impaired the binding of Tlp11-LBD to methyl pyruvate, and the MST measurement-derived binding curves of these mutants to methyl pyruvate were unable to obtain fitted *Kd* values (Figures 4B–D; Supplementary Figures S9A–C). Circular dichroism spectroscopy showed that a single mutation did not affect the secondary structures of L264A, N268A, I276A, Y291A, V318A, or T320A (Supplementary Figure S10), indicating that these residues are crucial for binding to methyl pyruvate. Compared to pyruvate, the additional alkyl group of methyl pyruvate or ethyl pyruvate might form hydrophobic interactions with L264 and V318 (Figure 4A; Supplementary Figure S11), which could stabilize the binding inside the pocket.

### 3.8 Discovering the antagonists for *Campylobacter jejuni* chemoreceptor Tlp11

**Figure 5:**
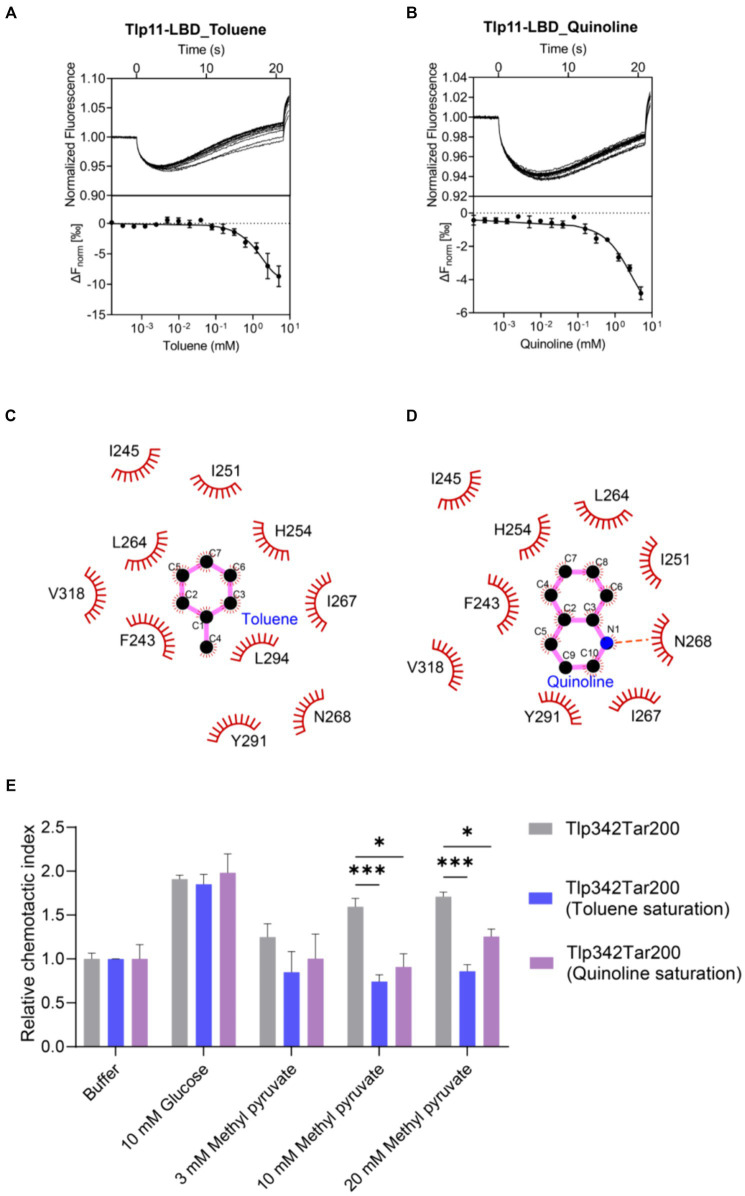
Detection of the antagonistic effects of toluene and quinoline. **(A,B)** MST measurements for the interactions of Tlp11-LBD with toluene **(A)** and quinoline **(B)**. The upper panel indicates the representative curves for thermophoresis of Tlp11-LBD with different concentrations of toluene or quinoline, while the lower panel indicates the dose–response curve with the fitting result. Error bars represent the standard errors of three independent replicates, shown as mean ± SD. The concentration for Tlp11-LBD was 250 nM, and the maximum concentration for the ligand was 5 mM, which was gradually diluted. **(C,D)** Binding interaction analysis between toluene **(C)** or quinoline **(D)** and Tlp11-LBD. Molecular docking was performed using AutoDock, and the interactions were illustrated using LigPlus. The hydrogen bond is represented by orange dashed lines. **(E)** The effects of toluene and quinoline on the chemotaxis of *E. coli* expressing the hybrid chemoreceptor Tlp342Tar200 to methyl pyruvate. *E. coli* VS188 cells expressing Tlp342Tar200 were adapted in 10 mM toluene or quinoline, and their chemotaxis to methyl pyruvate was measured. Significant differences, as compared to the Tlp342Tar200 (toluene or quinoline saturation), were calculated using a paired *t*-test; *p < 0.05 and ***p < 0.001.

Mutations on hydrophobic residues mentioned above greatly affected the binding of Tlp11-LBD to methyl pyruvate, indicating the importance of hydrophobic interactions for ligand binding. In addition, there are aromatic residues F243 and Y291 present in the membrane-proximal pocket of ATCC 33560 Tlp11-LBD (Supplementary Figure S12), which might form π–π interactions with aromatic molecules (Oshita and Shimazaki, 2022). We thus selected some aromatic compounds and detected their binding abilities to Tlp11-LBD using MST (Supplementary Table S4). Among these compounds, toluene and quinoline could bind directly to Tlp11-LBD, with *Kd* of 844 ± 172 μM and 905 ± 180 μM, respectively (Figures 5A,B). Molecular docking showed that both compounds might bind to the same membrane-proximal pocket as methyl pyruvate (Supplementary Figure S12). Toluene and quinoline were predicted to orientate similarly, and they might interact with additional hydrophobic residues compared to methyl pyruvate, including F243, I245, I251, and I267, while lose the interaction with T320 (Figures 5C,D; Supplementary Figure S12).

To explore the signal transduction induced by the binding of Tlp11 to toluene and quinoline, we first observed the chemotaxis of *E. coli* expressing the hybrid chemoreceptor Tlp342Tar200 towards these compounds. However, both toluene and quinoline failed to elicit a chemotactic response in a concentration-dependent manner (Supplementary Figures S13A,B). We then observed the chemotactic behavior of *C. jejuni* towards toluene and quinoline, but neither WT NCTC 11168 nor 11168ΩTlp11 cells exhibited chemotaxis towards them (Supplementary Figure S13C), indicating that toluene and quinoline act as the antagonists of Tlp11 and could not trigger the signal transmission.

To further ascertain the antagonistic function of toluene and quinoline, we performed competitive binding experiments with methyl pyruvate using MST. When Tlp11-LBD was saturated with 5 mM toluene or quinoline, the *Kd* of Tlp11-LBD interacting with methyl pyruvate increased to 18 ± 3 and 3.9 ± 1.1 mM, respectively (Supplementary Figures S14A,B), indicating that toluene and quinoline compete with the methyl pyruvate-binding pocket. We also measured the effects of toluene and quinoline on the chemotaxis of *E. coli* expressing Tlp342Tar200 to methyl pyruvate. When *E. coli* cells with Tlp342Tar200 were incubated in 10 mM toluene or quinoline, they lost the chemotaxis towards methyl pyruvate, while their responses to glucose were not affected (Figure 5E). All these results indicated that toluene and quinoline function as the antagonists by competitively binding to the methyl pyruvate-binding site in Tlp11-LBD.

### 3.9 Identification of Tlp11-LBD homologue proteins in bacteria that bind to methyl pyruvate

**Figure 6:**
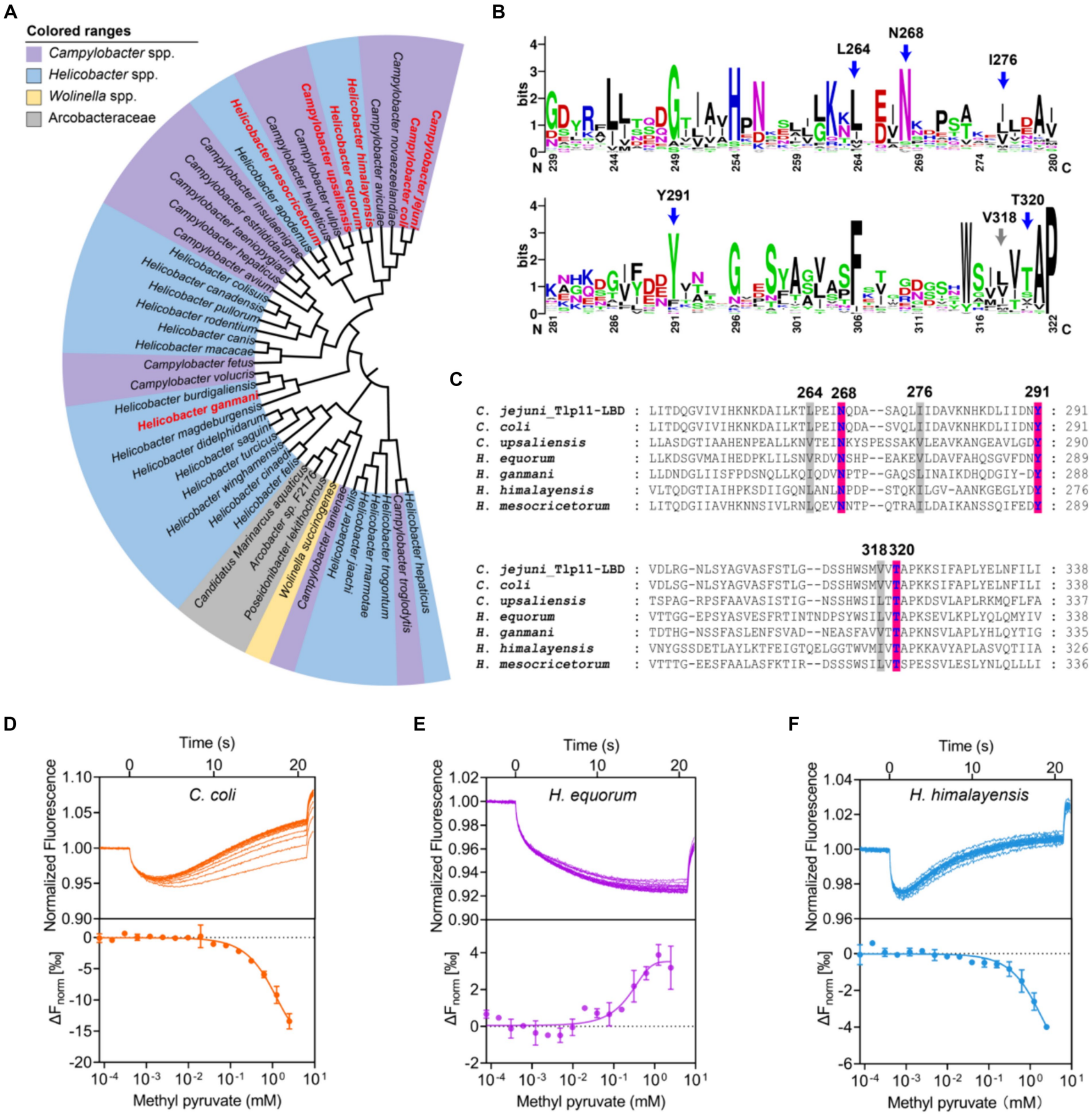
Identification of Tlp11-LBD homologues that bind to methyl pyruvate. **(A)** The phylogenetic tree showing the biological distribution of proteins with dCache domains that potentially bind to methyl pyruvate. The homologues selected for experimental verification are highlighted in red. **(B)** The conservation pattern found in Tlp11-LBD homologues. The sequence region corresponds to the membrane-proximal pocket of Tlp11-LBD (residues 239-322). The five crucial residues in Tlp11 that are present at the highest frequencies, L264, N268, I276, Y291, and T320, are indicated by blue arrows, while V318 is indicated by a grey arrow. **(C)** The sequence alignment of Tlp11-LBD homologues in *C. jejuni*, *C. coli*, *H. equorum*, *H. himalayensis*, *H. mesocricetorum*, *H. ganmani*, and *C. upsaliensis*. The red and grey areas indicate the key residues involved in the formation of hydrogen bonds and hydrophobic interactions, respectively, with methyl pyruvate. **(D–F)** The binding of Tlp11-LBD homologues in *C. coli* **(D)**, *H. equorum* **(E)**, and *H. himalayensis* **(F)** to methyl pyruvate, as measured using MST. Upper panel: thermophoresis raw data; lower panel: dose-response curve with the fitting result. Error bars represent the standard errors of three replicates. The concentration for the mutant protein was 250 nM, and the maximum concentration for the ligand was 2.5 mM, which was gradually diluted.

Analysis of 100 genomes from different *C. jejuni* strains in the NCBI Genome Database showed that Tlp11 is encoded by ~15% of the *C. jejuni* genomes (Supplementary Table S5), which is consistent with a previous report (Day et al., 2016). Next, we analyzed the distribution of Tlp11-LBD homologous proteins that may also bind to methyl pyruvate in other bacterial species. The Tlp11-LBD sequence (residues 32-332) from *C. jejuni* ATCC 33560 (NCBI accession number: AZU51669.1) was used as a query in a BLAST search against the NCBI RefSeq Database (Tatusova et al., 2014), and 44 sequences (including ATCC 33560 Tlp11) from different species with e-values less than e^−5^ were obtained (Supplementary Table S6). The identity of these sequences with those of *C. jejuni* Tlp11-LBD was >22% (Supplementary Table S6). We found that these potential Tlp11-LBD homologues mainly exist in the order Campylobacterales, including species from the host-associated genera *Campylobacter*, *Helicobacter*, *Wolinella*, and the family Arcobacteraceae (Figure 6A). The distribution of key residues for methyl pyruvate-binding in the active pocket of Tlp11 was analyzed in these 44 protein sequences by means of alignment, which revealed the presence of five crucial residues L264, N268, I276, Y291, and T320 in Tlp11 at the highest frequencies for binding to methyl pyruvate (Figure 6B). Two protein sequences contained all six key residues, while totally 21 sequences contained the conserved hydrophilic residues N268, Y291, and T320, which are distributed in the genera *Campylobacter* and *Helicobacter* (Supplementary Figure S15). All 44 proteins were chemoreceptors.

To evaluate whether these potential Tlp11-LBD homologues could bind to methyl pyruvate, we conducted a structural analysis of the LBDs of six chemoreceptors from *C. coli*, *H. equorum*, *H. himalayensis*, *H. mesocricetorum*, *H. ganmani*, and *C. upsaliensis* using Alphafold 2. All of these LBDs are dCache structures, and the spatial orientations of N, Y, and T corresponding to Tlp11-N268Y291T320, which is involved in the binding of methyl pyruvate in the membrane-proximal pocket, are similar to those of Tlp11-LBD (Figure 6C; Supplementary Figures S16A–F). Assessment of the binding abilities of these homologues to methyl pyruvate using MST showed that the chemoreceptors of all these 6 LBDs from *C. coli*, *H. equorum*, *H. himalayensis*, *H. mesocricetorum*, *H. ganmani*, and *C. upsaliensis* had the ability to bind to methyl pyruvate, with *Kd* values of 1.4 ± 0.5 mM, 340 ± 43 μM, 1.1 ± 0.3 mM, 1.2 ± 0.4 mM, 1.6 ± 0.2 mM, and 1.9 ± 0.5 mM, respectively (Figures 6D–F; Supplementary Figures S17A–C). These results demonstrated that chemoreceptors with a dCache domain that bind to methyl pyruvate are conserved in the order Campylobacterales.

## 4 Discussion

Methyl pyruvate is a widely used pharmaceutical and pesticide intermediate (Robinson et al., 1969; Tagashira et al., 2014) that has recently been detected in human blood as well (Barupal and Fiehn, 2019). This compound performs important functions in both prokaryotic and eukaryotic organisms. It serves as a carbon source for some bacteria and is metabolized by *Francisella noatunensis* subsp. *orientalis* (Ramírez-Paredes et al., 2017), improves the growth of *C. jejuni* by acting as a donor for the tricarboxylic acid cycle, and restores the persister cells of *E. coli* O157:H7 by stimulating metabolism as the sole carbon source (Chen et al., 2021). In some eukaryotic cells, methyl pyruvate supplies intramitochondrial pyruvate, reduces glutamate metabolism through glutamate dehydrogenase, and improves glutamate metabolism through alanine aminotransferase (to control the acid–base balance) (Oliver et al., 2010). Moreover, in rat pancreatic islets, methyl pyruvate is the substrate of lactate dehydrogenase and alanine aminotransferase, and can be converted directly into amino acids for cell utilization, displaying a higher metabolic efficiency than pyruvate (Jijakli et al., 1996). Notably, lactate dehydrogenase and alanine aminotransferase are also encoded by *C. jejuni* NCTC 11168 (NCBI accession numbers: CAL35282.1 and CAL34321.1) and ATCC 33560 (NCBI accession numbers: AZU50912.1 and AZU51579.1). It is also possible that methyl pyruvate is converted into other amino acids by these enzymes in *C. jejuni*. Similar growth responses were observed in *C. jejuni* with either methyl pyruvate or pyruvate added into the culture medium. Methyl pyruvate may serve as a precursor for pyruvate, but the mechanism by which methyl pyruvate conversion to pyruvate is still unclear.

Here we discovered novel ligands of *C. jejuni* Tlp11. To the best of our knowledge, Tlp11 is the first reported chemoreceptor that directly binds and senses methyl pyruvate. There are a number of reports showed that chemotactic behavior could be observed even in situations with binding affinity of *Kd* ~ 10^−4^–10^−2^ M (Martín-Mora et al., 2016; Khan et al., 2020; Chen et al., 2022), due to chemotactic systems amplifying the sensed external signals. The chemotactic response at high concentrations of chemicals might enable bacteria to search for benefit levels of attractants or to avoid harmful levels of repellents in the environment, which was also suggested by a previous report (Lopes and Sourjik, 2018). In our study, there is a pronounced correlation between the concentrations of methyl pyruvate that elicited attractant responses and the concentrations that promoted the growth of *C. jejuni*.

The dCache domains recognize diverse compounds, including amino acids, organic acids, polyamines, purines, and autoinducer-2 (Matilla et al., 2022, 2023b). However, this is the first report to show that the dCache domain binds methyl pyruvate, toluene, and quinoline, thus expanding the known range of ligands recognized by the dCache family. Considering that the residues for recognizing toluene and quinoline have some differences from those for methyl pyruvate, it would be meaningful to explore why the binding of toluene and quinoline could not trigger chemotactic signal. Chemoreceptors that bind ligands in the membrane-proximal subdomains have been reported in *Helicobacter pylori* TlpC and *C. jejuni* Tlp1 (Machuca et al., 2017; Duan et al., 2023). For the vast majority of dCache domains, ligands bind to the membrane-distal subdomain (Gavira et al., 2018; Khan et al., 2020). It would be interesting to further screen for ligand binding to the membrane-distal subdomain of Tlp11 and explore how the two subdomains work together to transmit signals.

Nutrient access is the primary benefit of bacterial chemotaxis. Previous studies have reported that chemotaxis is important for the pathogenesis and colonization of some intestinal pathogens (Takata et al., 1992; Chandrashekhar et al., 2017; Matilla et al., 2023a), and the ability to metabolize specific nutrients enhances the colonization of pathogens to specific tissues (Hofreuter et al., 2008). Chemotaxis is important for the colonization and infection of some invasive *C. jejuni* strains, including NCTC 11168, although it does not encode Tlp11 (Day et al., 2016; Korolik, 2019). The presence of Tlp11 in a few highly virulent *C. jejuni* strains would expand the chemoeffector spectrum of chemotaxis system for these strains. This might benefit the Tlp11-containing strains from some aspects, including the improvement of growth, as we showed here for the strain ATCC33560. In addition, the presence of Tlp11 was reported to have a significant influence on the adhesion and colonization of *C. jejuni* strain 520 with Tlp11 (Day et al., 2016), indicating the role of Tlp11-dependent chemotaxis in enhancing the virulence of *C. jejuni* strains with Tlp11. Blood is a potential source of nutrients for damaged tissues. The specific chemoattractant gradients present in inflamed and injured host tissues, including blood, enable pathogens to perform chemoattraction at sites of host injury (Zhou et al., 2023). Considering that methyl pyruvate can be detected in human blood, the evolution of Tlp11 might make *C. jejuni* attractive to methyl pyruvate derived from the blood, leading to the aggregation of *C. jejuni* at the sites of injury and impairing recovery.

Discovering direct-binding ligands and exploring their signaling properties are of great importance for studying the physiological functions of chemoreceptors. To verify that methyl pyruvate specifically stimulates transmembrane signaling via Tlp11-LBD, we constructed chimeras. In addition to designing the hybrid chemoreceptor Tlp11-Tar, which is a powerful tool for exploring the ligand specificity of the target LBD (Bi et al., 2016; Duan et al., 2023), we constructed the hybrid kinase Tlp11-PhoQ, by reasonably fusing the *E. coli* PhoQ cytoplasmic region with *C. jejuni* Tlp11-LBD. This hybrid kinase exhibited obvious responses upon stimulation with methyl pyruvate, with significant fluorescence enhancement controlled by the promoter of *mgtLA*. These chimeras can be used to verify the signaling molecules of bacterial receptors and explore the function of transmembrane signaling of the target LBD upon ligand binding.

To date, there have been very few reports on the natural functions of chemotaxis antagonists. As chemotaxis is an important virulence factor of pathogenic bacteria, inhibiting chemotactic signaling would be an effective strategy for preventing diseases. We previously reported the first example of the antagonist for *E. coli* chemoreceptor Tar, which significantly interfered Tar mediated chemotaxis towards the attractants (Bi et al., 2013). A recent study found that glucosamine, as a chemotaxis antagonist of *H. pylori* chemoreceptor TlpA, prevented the chemotaxis response to chemoattractant ligands and acted to block ligand binding (Johnson et al., 2021). The antagonistic effect on chemotaxis could also be achieved through periplasmic ligand-binding proteins. An antagonist of periplasmic glucose/galactose-binding protein that blocked *E. coli* chemotaxis to glucose via chemoreceptor Trg was discovered (Borrok et al., 2009).

Currently, the primary strategy for treating infections of pathogenic bacteria is the use of antibiotics. However, with the emergence of antibiotic resistance, multidrug-resistant strains have led to severe outcomes, including longer illness duration (Whitehouse et al., 2018). As sensing of environmental signals is related to host colonization and the pathogenicity, the inhibition of bacterial sensory systems might constitute a promising alternative approach for the treatment of diseases (Christensen et al., 2013; Taylor et al., 2022). Clinical studies have shown that omeprazole, an antibacterial medicine that disorients chemotactic bacteria, can increase the eradication rate of *H. pylori* from 25 to 95% upon combination with amoxicillin and clarithromycin (Matilla and Krell, 2023; Zhou et al., 2023). Designing functional inhibitors that interfere with environmental sensing and signaling via signal transduction proteins may be a new strategy for disease prevention and control. Therefore, considering the presence of Tlp11 in highly infective *C. jejuni* strains, designing inhibitors based on the backbones of toluene and quinoline that can hinder methyl pyruvate chemotaxis, via Tlp11, may be a fresh idea for prevention of *C. jejuni* infection and campylobacteriosis.

## 5 Conclusion

The chemotaxis is an important virulence factor for the food pathogen *C. jejuni*, and the core link among chemotaxis and pathogenicity lies in the discovery of signaling molecules sensed by chemoreceptors. Here, we identified a set of novel direct-binding ligands of chemoreceptor Tlp11, including the attractant methyl pyruvate that promoted *C. jejuni* growth and antagonists toluene and quinoline. By assessing the signaling properties of some constructed receptor chimeras, we proved that methyl pyruvate triggers transmembrane signaling via binding to Tlp11-LBD. Bioinformatics and experiments showed that the dCache domains with methyl pyruvate-binding sites are distributed in different host-related genera. Our work provides important insights into the mechanism of microbial chemotaxis towards methyl pyruvate and will facilitate further investigations into the fitness benefit of chemotaxis in growth and virulence. The interference of methyl pyruvate chemotaxis of *C. jejuni* may be a new strategy for preventing the chemotaxis and infection of this foodborne pathogen.

## Data availability statement

The original contributions presented in the study are included in the article/Supplementary material, further inquiries can be directed to the corresponding author.

## Author contributions

QZ: Data curation, Formal analysis, Methodology, Resources, Software, Validation, Visualization, Writing – original draft, Writing – review & editing. FY: Data curation, Methodology, Validation, Visualization, Writing – review & editing. WL: Data curation, Formal analysis, Methodology, Validation, Visualization, Writing – review & editing. SL: Investigation, Methodology, Supervision, Validation, Visualization, Writing – review & editing. SB: Conceptualization, Funding acquisition, Investigation, Methodology, Project administration, Resources, Supervision, Validation, Visualization, Writing – review & editing.

## Footnotes

^1^ https://www.ebi.ac.uk/Tools/msa/clustalo/

^2^ https://weblogo.berkeley.edu/logo.cgi

## References

[ref1] Barupal D. K. Fiehn O. (2019). Generating the blood exposome database using a comprehensive text mining and database fusion approach. Environ. Health Perspect. 127:97008. doi: 10.1289/ehp4713, PMID: 31557052 PMC6794490

[ref2] Bi S. Pollard A. M. Yang Y. Jin F. Sourjik V. (2016). Engineering hybrid chemotaxis receptors in bacteria. ACS Synth. Biol. 5, 989–1001. doi: 10.1021/acssynbio.6b00053, PMID: 27285081

[ref3] Bi S. Sourjik V. (2018). Stimulus sensing and signal processing in bacterial chemotaxis. Curr. Opin. Microbiol. 45, 22–29. doi: 10.1016/j.mib.2018.02.002, PMID: 29459288

[ref4] Bi S. Yu D. Si G. Luo C. Li T. Ouyang Q.. (2013). Discovery of novel chemoeffectors and rational design of Escherichia coli chemoreceptor specificity. Proc. Natl. Acad. Sci. USA 110, 16814–16819. doi: 10.1073/pnas.1306811110, PMID: 24082101 PMC3801017

[ref5] Borrok M. J. Zhu Y. Forest K. T. Kiessling L. L. (2009). Structure-based design of a periplasmic binding protein antagonist that prevents domain closure. ACS Chem. Biol. 4, 447–456. doi: 10.1021/cb900021q, PMID: 19348466 PMC2742562

[ref6] Braga C. Travis K. P. (2006). Configurational constant pressure molecular dynamics. J. Chem. Phys. 124:104102. doi: 10.1063/1.2172601 16542063

[ref7] Briegel A. Li X. Bilwes A. M. Hughes K. T. Jensen G. J. Crane B. R. (2012). Bacterial chemoreceptor arrays are hexagonally packed trimers of receptor dimers networked by rings of kinase and coupling proteins. Proc. Natl. Acad. Sci. USA 109, 3766–3771. doi: 10.1073/pnas.1115719109, PMID: 22355139 PMC3309718

[ref8] Bussi G. Donadio D. Parrinello M. (2007). Canonical sampling through velocity rescaling. J. Chem. Phys. 126:014101. doi: 10.1063/1.2408420, PMID: 17212484

[ref9] Chandrashekhar K. Gangaiah D. Pina-Mimbela R. Kassem I. Jeon B. H. Rajashekara G. (2015). Transducer like proteins of Campylobacter jejuni 81-176: role in chemotaxis and colonization of the chicken gastrointestinal tract. Front. Cell. Infect. Microbiol. 5:46. doi: 10.3389/fcimb.2015.00046, PMID: 26075188 PMC4444964

[ref10] Chandrashekhar K. Kassem I. Rajashekara G. (2017). Campylobacter jejuni transducer like proteins: chemotaxis and beyond. Gut Microbes 8, 323–334. doi: 10.1080/19490976.2017.1279380, PMID: 28080213 PMC5570417

[ref11] Chen X. Bi S. Ma X. Sourjik V. Lai L. (2022). Discovery of a new chemoeffector for Escherichia coli chemoreceptor Tsr and identification of a molecular mechanism of repellent sensing. ACS Bio Med Chem Au 2, 386–394. doi: 10.1021/acsbiomedchemau.1c00055, PMID: 37102165 PMC10125284

[ref12] Chen H. Green A. Martz K. Wu X. Alzahrani A. Warriner K. (2021). The progress of type II persisters of Escherichia coli O157:H7 to a non-culturable state during prolonged exposure to antibiotic stress with revival being aided through acid-shock treatment and provision of methyl pyruvate. Can. J. Microbiol. 67, 518–528. doi: 10.1139/cjm-2020-0339, PMID: 33125853

[ref13] Christensen Q. H. Grove T. L. Booker S. J. Greenberg E. P. (2013). A high-throughput screen for quorum-sensing inhibitors that target acyl-homoserine lactone synthases. Proc. Natl. Acad. Sci. USA 110, 13815–13820. doi: 10.1073/pnas.1313098110, PMID: 23924613 PMC3752275

[ref14] Colin R. Ni B. Laganenka L. Sourjik V. (2021). Multiple functions of flagellar motility and chemotaxis in bacterial physiology. FEMS Microbiol. Rev. 45, 1–19. doi: 10.1093/femsre/fuab038, PMID: 34227665 PMC8632791

[ref15] Day C. J. Hartley-Tassell L. E. Shewell L. K. King R. M. Tram G. Day S. K.. (2012). Variation of chemosensory receptor content of Campylobacter jejuni strains and modulation of receptor gene expression under different in vivo and in vitro growth conditions. BMC Microbiol. 12:128. doi: 10.1186/1471-2180-12-128, PMID: 22747654 PMC3461409

[ref16] Day C. J. King R. M. Shewell L. K. Tram G. Najnin T. Hartley-Tassell L. E.. (2016). A direct-sensing galactose chemoreceptor recently evolved in invasive strains of Campylobacter jejuni. Nat. Commun. 7:13206. doi: 10.1038/ncomms13206, PMID: 27762269 PMC5080441

[ref17] Duan J. Zhao Q. Wang Y. Chi Z. Li W. Wang X.. (2023). The dCache domain of the chemoreceptor Tlp1 in Campylobacter jejuni binds and triggers chemotaxis toward formate. mBio 14:e356422. doi: 10.1128/mbio.03564-22, PMID: 37052512 PMC10294657

[ref18] Elgamoudi B. A. Andrianova E. P. Shewell L. K. Day C. J. King R. M. Taha R. H.. (2021). The Campylobacter jejuni chemoreceptor Tlp10 has a bimodal ligand-binding domain and specificity for multiple classes of chemoeffectors. Sci. Signal. 14:eabc8521. doi: 10.1126/scisignal.abc8521 33402336 PMC8112392

[ref19] Feng H. Lv Y. Krell T. Fu R. Liu Y. Xu Z.. (2022). Signal binding at both modules of its dCache domain enables the McpA chemoreceptor of Bacillus velezensis to sense different ligands. Proc. Natl. Acad. Sci. USA 119:e2201747119. doi: 10.1073/pnas.2201747119, PMID: 35858353 PMC9303924

[ref20] Fernández M. Ortega Á. Rico-Jiménez M. Martín-Mora D. Daddaoua A. Matilla M. A.. (2018). High-throughput screening to identify chemoreceptor ligands. Methods Mol. Biol. 1729, 291–301. doi: 10.1007/978-1-4939-7577-8_23 29429099

[ref21] Filipski A. Murillo O. Freydenzon A. Tamura K. Kumar S. (2014). Prospects for building large timetrees using molecular data with incomplete gene coverage among species. Mol. Biol. Evol. 31, 2542–2550. doi: 10.1093/molbev/msu200, PMID: 24974376 PMC4137717

[ref22] Gao M. He Y. Yin X. Zhong X. Yan B. Wu Y.. (2021). Ca^2+^ sensor-mediated ROS scavenging suppresses rice immunity and is exploited by a fungal effector. Cell 184, 5391–5404.e5317. doi: 10.1016/j.cell.2021.09.009 34597584

[ref23] Gavira J. A. Ortega Á. Martín-Mora D. Conejero-Muriel M. T. Corral-Lugo A. Morel B.. (2018). Structural basis for polyamine binding at the dCACHE domain of the McpU chemoreceptor from Pseudomonas putida. J. Mol. Biol. 430, 1950–1963. doi: 10.1016/j.jmb.2018.05.008, PMID: 29758259

[ref24] Guerry P. Ewing C. P. Schirm M. Lorenzo M. Kelly J. Pattarini D.. (2006). Changes in flagellin glycosylation affect Campylobacter autoagglutination and virulence. Mol. Microbiol. 60, 299–311. doi: 10.1111/j.1365-2958.2006.05100.x, PMID: 16573682 PMC1424674

[ref25] Hartley-Tassell L. E. Shewell L. K. Day C. J. Wilson J. C. Sandhu R. Ketley J. M.. (2010). Identification and characterization of the aspartate chemosensory receptor of Campylobacter jejuni. Mol. Microbiol. 75, 710–730. doi: 10.1111/j.1365-2958.2009.07010.x 20025667

[ref26] Hazelbauer G. L. Falke J. J. Parkinson J. S. (2008). Bacterial chemoreceptors: high-performance signaling in networked arrays. Trends Biochem. Sci. 33, 9–19. doi: 10.1016/j.tibs.2007.09.014, PMID: 18165013 PMC2890293

[ref27] Hazeleger W. C. Wouters J. A. Rombouts F. M. Abee T. (1998). Physiological activity of Campylobacter jejuni far below the minimal growth temperature. Appl. Environ. Microbiol. 64, 3917–3922. doi: 10.1128/aem.64.10.3917-3922.1998, PMID: 9758819 PMC106578

[ref28] Hess B. (2008). P-LINCS: a parallel linear constraint solver for molecular simulation. J. Chem. Theory Comput. 4, 116–122. doi: 10.1021/ct700200b, PMID: 26619985

[ref29] Hofreuter D. Novik V. Galán J. E. (2008). Metabolic diversity in Campylobacter jejuni enhances specific tissue colonization. Cell Host Microbe 4, 425–433. doi: 10.1016/j.chom.2008.10.002, PMID: 18996343

[ref30] Jijakli H. Nadi A. B. Cook L. Best L. Sener A. Malaisse W. J. (1996). Insulinotropic action of methyl pyruvate: enzymatic and metabolic aspects. Arch. Biochem. Biophys. 335, 245–257. doi: 10.1006/abbi.1996.0505, PMID: 8914921

[ref31] Johnson K. S. Elgamoudi B. A. Jen F. E. Day C. J. Sweeney E. G. Pryce M. L.. (2021). The dCache chemoreceptor TlpA of Helicobacter pylori binds multiple attractant and antagonistic ligands via distinct sites. MBio 12:e0181921. doi: 10.1128/mBio.01819-21, PMID: 34340539 PMC8406319

[ref32] Jumper J. Evans R. Pritzel A. Green T. Figurnov M. Ronneberger O.. (2021). Highly accurate protein structure prediction with AlphaFold. Nature 596, 583–589. doi: 10.1038/s41586-021-03819-2, PMID: 34265844 PMC8371605

[ref33] Khan M. F. Machuca M. A. Rahman M. M. Koç C. Norton R. S. Smith B. J.. (2020). Structure-activity relationship study reveals the molecular basis for specific sensing of hydrophobic amino acids by the Campylobacter jejuni chemoreceptor Tlp3. Biomol. Ther. 10:744. doi: 10.3390/biom10050744, PMID: 32403336 PMC7277094

[ref34] Kim S. H. Wang W. Kim K. K. (2002). Dynamic and clustering model of bacterial chemotaxis receptors: structural basis for signaling and high sensitivity. Proc. Natl. Acad. Sci. USA 99, 11611–11615. doi: 10.1073/pnas.132376499, PMID: 12186970 PMC129317

[ref35] Konkel M. E. Joens L. A. (1990). Effect of enteroviruses on adherence to and invasion of HEp-2 cells by Campylobacter isolates. Infect. Immun. 58, 1101–1105. doi: 10.1128/iai.58.4.1101-1105.1990, PMID: 2156779 PMC258588

[ref36] Korolik V. (2019). The role of chemotaxis during Campylobacter jejuni colonisation and pathogenesis. Curr. Opin. Microbiol. 47, 32–37. doi: 10.1016/j.mib.2018.11.001, PMID: 30476739

[ref37] Lanave C. Licciulli F. De Robertis M. Marolla A. Attimonelli M. (2002). Update of AMmtDB: a database of multi-aligned Metazoa mitochondrial DNA sequences. Nucleic Acids Res. 30, 174–175. doi: 10.1093/nar/30.1.174, PMID: 11752285 PMC99074

[ref38] Lange F. Pfennigwerth N. Höfken L. M. Gatermann S. G. Kaase M. (2019). Characterization of mutations in Escherichia coli PBP2 leading to increased carbapenem MICs. J. Antimicrob. Chemother. 74, 571–576. doi: 10.1093/jac/dky476 30496417

[ref39] Letunic I. Bork P. (2021). Interactive tree of life (iTOL) v5: an online tool for phylogenetic tree display and annotation. Nucleic Acids Res. 49, W293–W296. doi: 10.1093/nar/gkab301, PMID: 33885785 PMC8265157

[ref40] Li R. Li A. Zhang Y. Fu J. (2023). The emerging role of recombineering in microbiology. Eng. Microbiol. 3:100097. doi: 10.1016/j.engmic.2023.100097 PMC11610958 39628926

[ref41] Linke P. Amaning K. Maschberger M. Vallee F. Steier V. Baaske P.. (2016). An automated microscale thermophoresis screening approach for fragment-based lead discovery. J. Biomol. Screen. 21, 414–421. doi: 10.1177/1087057115618347, PMID: 26637553 PMC4800460

[ref42] Lopes J. G. Sourjik V. (2018). Chemotaxis of Escherichia coli to major hormones and polyamines present in human gut. ISME J. 12, 2736–2747. doi: 10.1038/s41396-018-0227-5, PMID: 29995838 PMC6194112

[ref43] Luu R. A. Schomer R. A. Brunton C. N. Truong R. Ta A. P. Tan W. A.. (2019). Hybrid two-component sensors for identification of bacterial chemoreceptor function. Appl. Environ. Microbiol. 85, e01626–e01619. doi: 10.1128/aem.01626-19, PMID: 31492670 PMC6821969

[ref44] Machuca M. A. Johnson K. S. Liu Y. C. Steer D. L. Ottemann K. M. Roujeinikova A. (2017). Helicobacter pylori chemoreceptor TlpC mediates chemotaxis to lactate. Sci. Rep. 7:14089. doi: 10.1038/s41598-017-14372-2 29075010 PMC5658362

[ref45] Maier J. A. Martinez C. Kasavajhala K. Wickstrom L. Hauser K. E. Simmerling C. (2015). ff14SB: improving the accuracy of protein side chain and backbone parameters from ff99SB. J. Chem. Theory Comput. 11, 3696–3713. doi: 10.1021/acs.jctc.5b00255, PMID: 26574453 PMC4821407

[ref46] Marchant J. Wren B. Ketley J. (2002). Exploiting genome sequence: predictions for mechanisms of Campylobacter chemotaxis. Trends Microbiol. 10, 155–159. doi: 10.1016/s0966-842x(02)02323-5, PMID: 11912013

[ref47] Martín-Mora D. Ortega Á. Pérez-Maldonado F. J. Krell T. Matilla M. A. (2018). The activity of the C4-dicarboxylic acid chemoreceptor of Pseudomonas aeruginosa is controlled by chemoattractants and antagonists. Sci. Rep. 8:2102. doi: 10.1038/s41598-018-20283-7, PMID: 29391435 PMC5795001

[ref48] Martín-Mora D. Ortega A. Reyes-Darias J. A. García V. López-Farfán D. Matilla M. A.. (2016). Identification of a chemoreceptor in Pseudomonas aeruginosa that specifically mediates chemotaxis toward α-ketoglutarate. Front. Microbiol. 7:1937. doi: 10.3389/fmicb.2016.01937, PMID: 27965656 PMC5126104

[ref49] Matilla M. A. Gavira J. A. Krell T. (2023a). Accessing nutrients as the primary benefit arising from chemotaxis. Curr. Opin. Microbiol. 75:102358. doi: 10.1016/j.mib.2023.102358, PMID: 37459734

[ref50] Matilla M. A. Krell T. (2018). The effect of bacterial chemotaxis on host infection and pathogenicity. FEMS Microbiol. Rev. 42, 40–67. doi: 10.1093/femsre/fux052 29069367

[ref51] Matilla M. A. Krell T. (2023). Targeting motility and chemotaxis as a strategy to combat bacterial pathogens. Microb. Biotechnol. 16, 2205–2211. doi: 10.1111/1751-7915.14306, PMID: 37387327 PMC10686171

[ref52] Matilla M. A. Monteagudo-Cascales E. Cerna-Vargas J. P. Gumerov V. M. Zhulin I. B. Krell T. (2023b). Is it possible to predict signal molecules that are recognized by bacterial receptors? Environ. Microbiol. 25, 11–16. doi: 10.1111/1462-2920.16143, PMID: 36054735 PMC9851934

[ref53] Matilla M. A. Velando F. Martín-Mora D. Monteagudo-Cascales E. Krell T. (2022). A catalogue of signal molecules that interact with sensor kinases, chemoreceptors and transcriptional regulators. FEMS Microbiol. Rev. 46, 1–31. doi: 10.1093/femsre/fuab043, PMID: 34424339

[ref54] Mund N. L. Masanta W. O. Goldschmidt A. M. Lugert R. Groß U. Zautner A. E. (2016). Association of Campylobacter jejuni ssp. jejuni chemotaxis receptor genes with multilocus sequence types and source of isolation. Eur J Microbiol Immunol 6, 162–177. doi: 10.1556/1886.2015.00041, PMID: 27766165 PMC5063009

[ref55] Neumann S. Grosse K. Sourjik V. (2012). Chemotactic signaling via carbohydrate phosphotransferase systems in Escherichia coli. Proc. Natl. Acad. Sci. USA 109, 12159–12164. doi: 10.1073/pnas.1205307109, PMID: 22778402 PMC3409764

[ref56] O’brien S. J. (2017). The consequences of Campylobacter infection. Curr. Opin. Gastroenterol. 33, 14–20. doi: 10.1097/mog.0000000000000329 27798443

[ref57] Oliver R. Friday E. Turturro F. Welbourne T. (2010). Troglitazone regulates anaplerosis via a pull/push affect on glutamate dehydrogenase mediated glutamate deamination in kidney-derived epithelial cells; implications for the Warburg effect. Cell. Physiol. Biochem. 26, 619–628. doi: 10.1159/000322329 21063099

[ref58] Ortega Á. Zhulin I. B. Krell T. (2017). Sensory repertoire of bacterial chemoreceptors. Microbiol. Mol. Biol. Rev. 81, e00033–e00017. doi: 10.1128/mmbr.00033-17, PMID: 29070658 PMC5706747

[ref59] Oshita H. Shimazaki Y. (2022). π-π stacking interaction of metal phenoxyl radical complexes. Molecules 27:1135. doi: 10.3390/molecules27031135, PMID: 35164397 PMC8840625

[ref60] Parkinson J. S. Hazelbauer G. L. Falke J. J. (2015). Signaling and sensory adaptation in Escherichia coli chemoreceptors: 2015 update. Trends Microbiol. 23, 257–266. doi: 10.1016/j.tim.2015.03.003, PMID: 25834953 PMC4417406

[ref61] Ramírez-Paredes J. G. Thompson K. D. Metselaar M. Shahin K. Soto E. Richards R. H.. (2017). A polyphasic approach for phenotypic and genetic characterization of the fastidious aquatic pathogen Francisella noatunensis subsp. orientalis. Front. Microbiol. 8:2324. doi: 10.3389/fmicb.2017.02324, PMID: 29312155 PMC5733052

[ref62] Riechmann C. Zhang P. (2023). Recent structural advances in bacterial chemotaxis signalling. Curr. Opin. Struct. Biol. 79:102565. doi: 10.1016/j.sbi.2023.102565, PMID: 36868078 PMC10460253

[ref63] Robinson H. J. Silber R. H. Graessle O. E. (1969). Thiabendazole: toxicological, pharmacological and antifungal properties. Tex. Rep. Biol. Med. 27:537. doi: 10.1038/icb.1970.33 4905215

[ref64] Sarkar A. Santoro J. Di Biasi L. Marrafino F. Piotto S. (2022). YAMACS: a graphical interface for GROMACS. Bioinformatics 38, 4645–4646. doi: 10.1093/bioinformatics/btac573, PMID: 35997557

[ref65] Seeliger D. De Groot B. L. (2010). Ligand docking and binding site analysis with PyMOL and Autodock/Vina. J. Comput. Aided Mol. Des. 24, 417–422. doi: 10.1007/s10822-010-9352-6 20401516 PMC2881210

[ref66] Si G. Yang W. Bi S. Luo C. Ouyang Q. (2012). A parallel diffusion-based microfluidic device for bacterial chemotaxis analysis. Lab Chip 12, 1389–1394. doi: 10.1039/c2lc21219f, PMID: 22361931

[ref67] Silva-Jiménez H. García-Fontana C. Cadirci B. H. Ramos-González M. I. Ramos J. L. Krell T. (2012). Study of the TmoS/TmoT two-component system: towards the functional characterization of the family of TodS/TodT like systems. Microb. Biotechnol. 5, 489–500. doi: 10.1111/j.1751-7915.2011.00322.x, PMID: 22212183 PMC3815326

[ref68] Somavanshi R. Ghosh B. Sourjik V. (2016). Sugar influx sensing by the phosphotransferase system of Escherichia coli. PLoS Biol. 14:e2000074. doi: 10.1371/journal.pbio.2000074, PMID: 27557415 PMC4996493

[ref69] Sourjik V. Vaknin A. Shimizu T. S. Berg H. C. (2007). In vivo measurement by FRET of pathway activity in bacterial chemotaxis. Methods Enzymol. 423, 365–391. doi: 10.1016/s0076-6879(07)23017-4, PMID: 17609141

[ref70] Sousa S. F. Fernandes P. A. Ramos M. J. (2006). Protein-ligand docking: current status and future challenges. Proteins 65, 15–26. doi: 10.1002/prot.21082, PMID: 16862531

[ref71] Tagashira H. Shinoda Y. Shioda N. Fukunaga K. (2014). Methyl pyruvate rescues mitochondrial damage caused by SIGMAR1 mutation related to amyotrophic lateral sclerosis. Biochim. Biophys. Acta 1840, 3320–3334. doi: 10.1016/j.bbagen.2014.08.012, PMID: 25175561

[ref72] Taha E. B. A. Andrianova E. P. Haselhorst T. Day C. J. Hartley-Tassell L. E. King R. M.. (2022). Diverse sensory repertoire of paralogous chemoreceptors Tlp2, Tlp3, and Tlp4 in Campylobacter jejuni. Microbiol Spectr 10:e0364622. doi: 10.1128/spectrum.03646-22, PMID: 36374080 PMC9769880

[ref73] Takata T. Fujimoto S. Amako K. (1992). Isolation of nonchemotactic mutants of Campylobacter jejuni and their colonization of the mouse intestinal tract. Infect. Immun. 60, 3596–3600. doi: 10.1128/iai.60.9.3596-3600.1992, PMID: 1500167 PMC257366

[ref74] Tatusova T. Ciufo S. Fedorov B. O'neill K. Tolstoy I. (2014). RefSeq microbial genomes database: new representation and annotation strategy. Nucleic Acids Res. 42, D553–D559. doi: 10.1093/nar/gkt1274, PMID: 24316578 PMC3965038

[ref75] Taylor I. R. Jeffrey P. D. Moustafa D. A. Goldberg J. B. Bassler B. L. (2022). The PqsE active site as a target for small molecule antimicrobial agents against Pseudomonas aeruginosa. Biochemistry 61, 1894–1903. doi: 10.1021/acs.biochem.2c00334, PMID: 35985643 PMC9454246

[ref76] Vegge C. S. Brøndsted L. Li Y. P. Bang D. D. Ingmer H. (2009). Energy taxis drives Campylobacter jejuni toward the most favorable conditions for growth. Appl. Environ. Microbiol. 75, 5308–5314. doi: 10.1128/aem.00287-09, PMID: 19542337 PMC2725471

[ref77] Wagley S. Newcombe J. Laing E. Yusuf E. Sambles C. M. Studholme D. J.. (2014). Differences in carbon source utilisation distinguish Campylobacter jejuni from Campylobacter coli. BMC Microbiol. 14:262. doi: 10.1186/s12866-014-0262-y, PMID: 25348335 PMC4219013

[ref78] Wang L. C. Morgan L. K. Godakumbura P. Kenney L. J. Anand G. S. (2012). The inner membrane histidine kinase EnvZ senses osmolality via helix-coil transitions in the cytoplasm. EMBO J. 31, 2648–2659. doi: 10.1038/emboj.2012.99, PMID: 22543870 PMC3365433

[ref79] Wassenaar T. M. Van Der Zeijst B. A. Ayling R. Newell D. G. (1993). Colonization of chicks by motility mutants of Campylobacter jejuni demonstrates the importance of flagellin a expression. J. Gen. Microbiol. 139, 1171–1175. doi: 10.1099/00221287-139-6-1171, PMID: 8360610

[ref80] Whitehouse C. A. Zhao S. Tate H. (2018). Antimicrobial resistance in Campylobacter species: mechanisms and genomic epidemiology. Adv. Appl. Microbiol. 103, 1–47. doi: 10.1016/bs.aambs.2018.01.001, PMID: 29914655

[ref81] Wuichet K. Zhulin I. B. (2010). Origins and diversification of a complex signal transduction system in prokaryotes. Sci. Signal. 3:ra50. doi: 10.1126/scisignal.2000724, PMID: 20587806 PMC3401578

[ref82] Yao R. Burr D. H. Guerry P. (1997). CheY-mediated modulation of Campylobacter jejuni virulence. Mol. Microbiol. 23, 1021–1031. doi: 10.1046/j.1365-2958.1997.2861650.x, PMID: 9076738

[ref83] Young K. T. Davis L. M. Dirita V. J. (2007). Campylobacter jejuni: molecular biology and pathogenesis. Nat. Rev. Microbiol. 5, 665–679. doi: 10.1038/nrmicro1718 17703225

[ref84] Yuan J. Jin F. Glatter T. Sourjik V. (2017). Osmosensing by the bacterial PhoQ/PhoP two-component system. Proc. Natl. Acad. Sci. USA 114, E10792–E10798. doi: 10.1073/pnas.1717272114, PMID: 29183977 PMC5740661

[ref85] Zhou B. Szymanski C. M. Baylink A. (2023). Bacterial chemotaxis in human diseases. Trends Microbiol. 31, 453–467. doi: 10.1016/j.tim.2022.10.007 36411201 PMC11238666

