# Supplementary material for: Identification of a dCache-type chemoreceptor in Campylobacter jejuni that specifically mediates chemotaxis towards methyl pyruvate
Source: Front Microbiol. 2024 May 9;15:1400284. doi: 10.3389/fmicb.2024.1400284 (PMC11111895; doi:10.3389/fmicb.2024.1400284)
Supplement: Supplementary file 1 [file Image_1.PDF]

Supplementary Figures

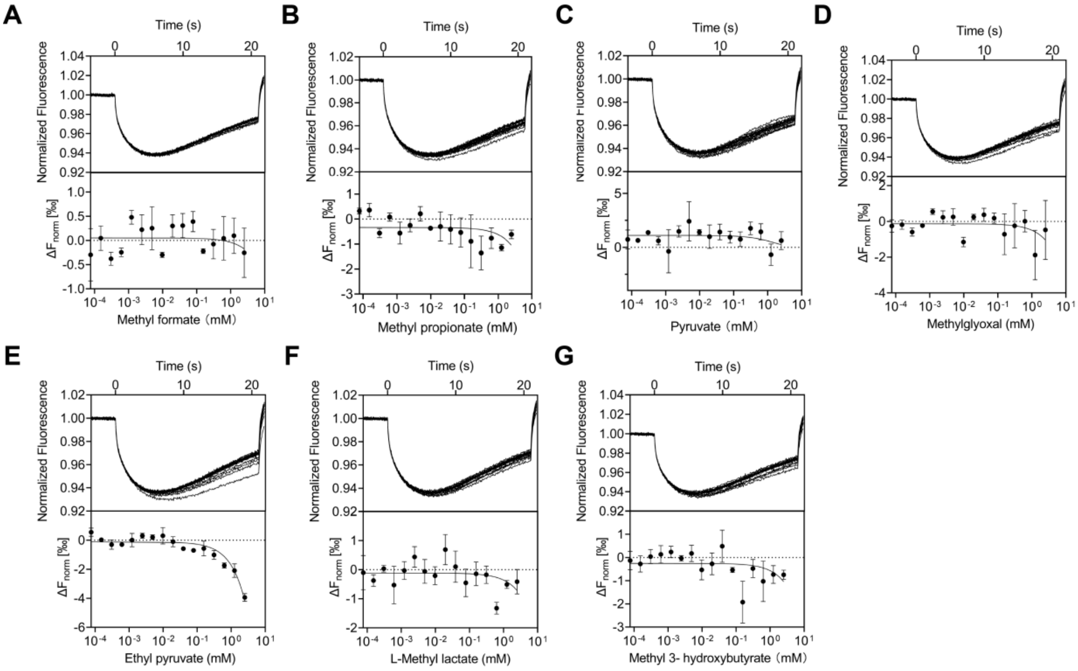

FIG S1 Binding analyses of Tlp11 with methyl pyruvate analogues. (A-H) MST measurements for the interactions of Tlp11-LBD with methyl formate (A), methyl propionate (B), pyruvate (C), methylglyoxal (D), ethyl pyruvate (E), L-methyl lactate (F) and methyl 3- hydroxybutyrate (G). The upper panel indicates the representative curves for thermophoresis of Tlp11-LBD proteins with different concentrations of compounds, while the lower panel indicates the dose-response curve with the fitting result. Error bars represent the standard errors of three independent replicates, shown as mean ± SD. The concentration for Tlp11-LBD proteins was 250 nM, and the maximum concentration for compounds was 2.5 mM, which was diluted gradually.

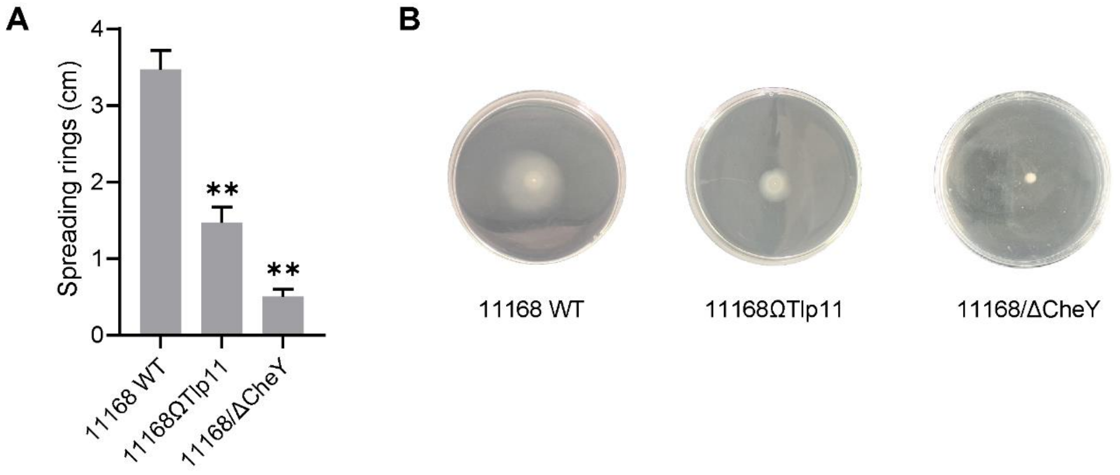

FIG S2 Measurements of the spreading of *C. jejuni* WT NCTC 11168, 11168ΩTlp11, and 11168ΔCheY. (A) The diameters of the spreading rings for WT NCTC 11168 strain, ΩTlp11, and ΔCheY strain inoculated into semi-solid MH soft agar. (B) The original representative results of the spreading rings for WT NCTC 11168 strain, ΩTlp11, and ΔCheY strain. Error bars indicate standard errors of three independent replicates, shown as mean ± SD. The *p*-values were calculated using the paired *t*-test; $^{**}p<0.01$, compared to 11168 WT.

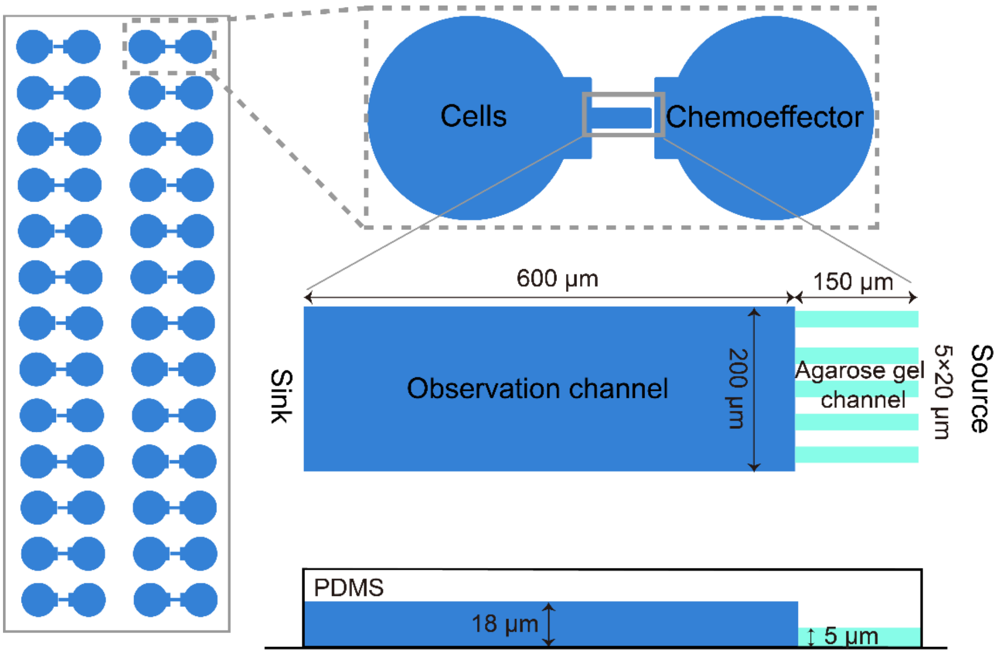

FIG S3 The microfluidic device used in this study. Each chip contains 24 parallel units, with each module containing a sink pore and a source pore. The sink pore and source pore are connected by 5 agarose channels (150 μm long, 20 μm wide, and 5 μm high) and an observation channel (600 μm long, 200 μm wide, and 18 μm high).

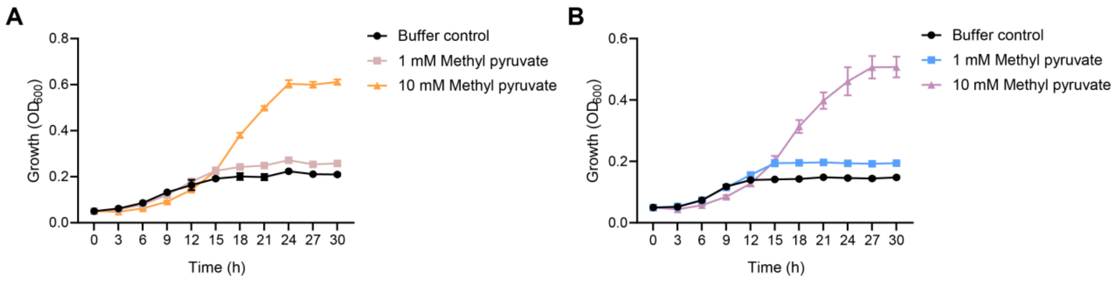

FIG S4 The growth curves of *C. jejuni* WT NCTC 11168 (A) and ΩTlp11 (B) in different concentrations of methyl pyruvate. The medium was supplemented with indicated concentrations of methyl pyruvate. In (A,B), the medium for *C. jejuni* growth was Minimal Essential Medium supplemented with fetal bovine serum. Error bars represent the standard errors of three independent replicates, shown as mean ± SD.

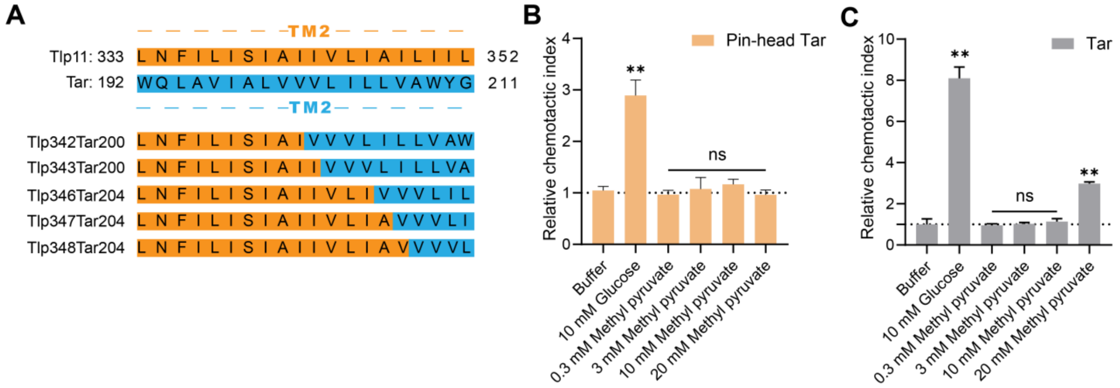

FIG S5 Sequence alignment for hybrid chemoreceptors and measurements of chemotaxis to methyl pyruvate using microfluidics. (A) Sequence alignment for Tlp11-TM2 and Tar-TM2, and the fusion sequences for part of Tlp342Tar200, Tlp343Tar200, Tlp346Tar204, Tlp347Tar204, and Tlp348Tar204 are shown below. (B–C) The relative chemotactic index of *E. coli* VS188 cells expressing pin-head Tar (B), or Tar (C) as the sole receptor, in response to the indicated concentrations of methyl pyruvate or buffer at 50 min. Error bars indicate the standard errors of three independent replicates, shown as mean ± SD. The *p*-values were calculated using the paired *t*-test; ***p*<0.01. The "ns" means no significant difference compared to buffer.

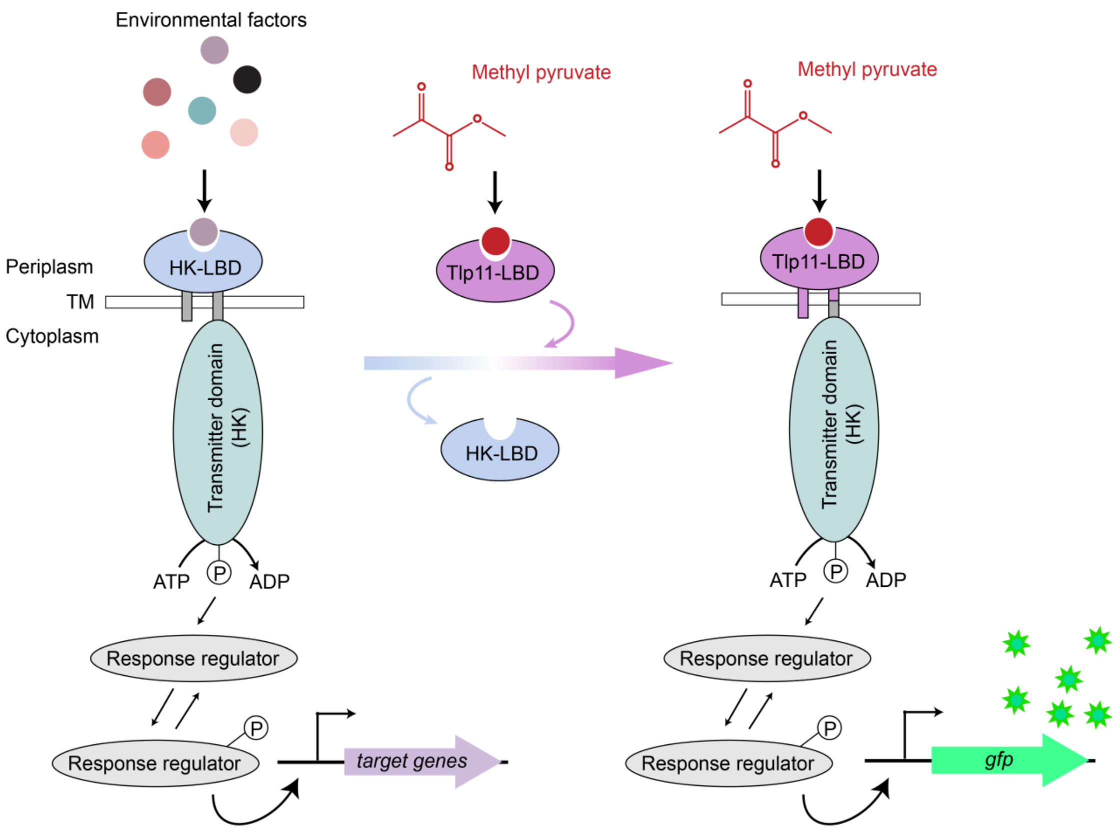

FIG S6 Design of the TCS for sensing methyl pyruvate. The LBD of HK was replaced by Tlp11-LBD and fused in the TM2. The *gfp* was placed under the promoter regulated by the engineered TCS.

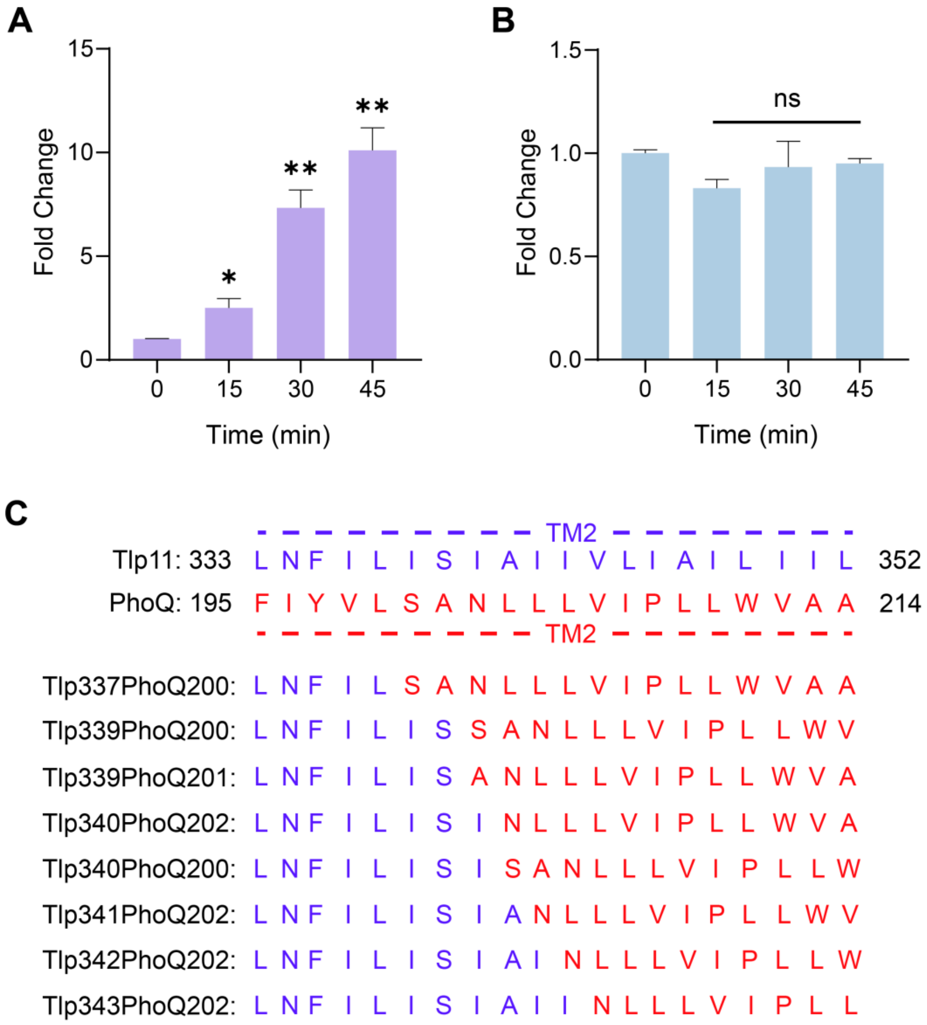

FIG S7 The responses of PhoQ-PhoP-GFP and EnvZ-OmpR-GFP systems and construction of Tlp11-PhoQ hybrid kinases. (A) Expression of GFP reporter under control of *mgtLA* promoter upon an osmotic upshift induced by 400 mM NaCl. (B) Expression of GFP reporter under control of *ompC* promoter upon an osmotic upshift induced by 20% sucrose. In (A, B), the values were normalized to the fluorescent signal at time point 0. (C) Sequence alignment for Tlp11-TM2 and PhoQ-TM2, and part of the fusion sequences for Tlp337PhoQ200, Tlp339PhoQ200, Tlp339PhoQ201, Tlp340PhoQ202, Tlp340PhoQ200, Tlp341PhoQ202, Tlp342PhoQ202, and Tlp343PhoQ202 are shown below. Error bars indicate the standard errors of three independent replicates, shown as mean ± SD. The *p*-values were calculated using the paired *t*-test; *$p<0.05$, **$p<0.01$. The "ns" means no significant difference compared to the time point 0.

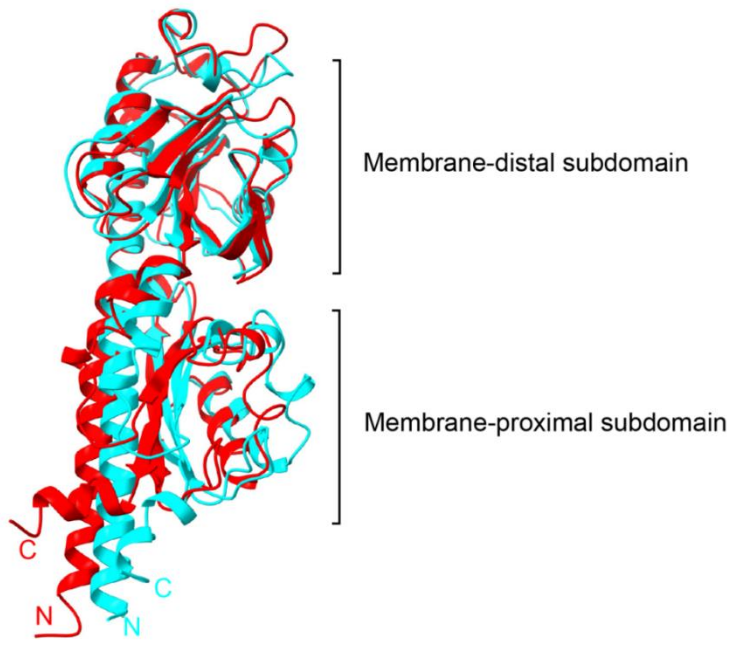

FIG S8 MD simulations for Tlp11-LBD structure. Overlay diagram of the initial protein conformation predicted using AlphaFold 2 (Jumper et al., 2021) (cyan) and optimized conformation (red) after 100 ns MD simulations. The N- and C- terminal as well as the membrane-proximal and membrane-distal subdomains of Tlp11-LBD were labeled.

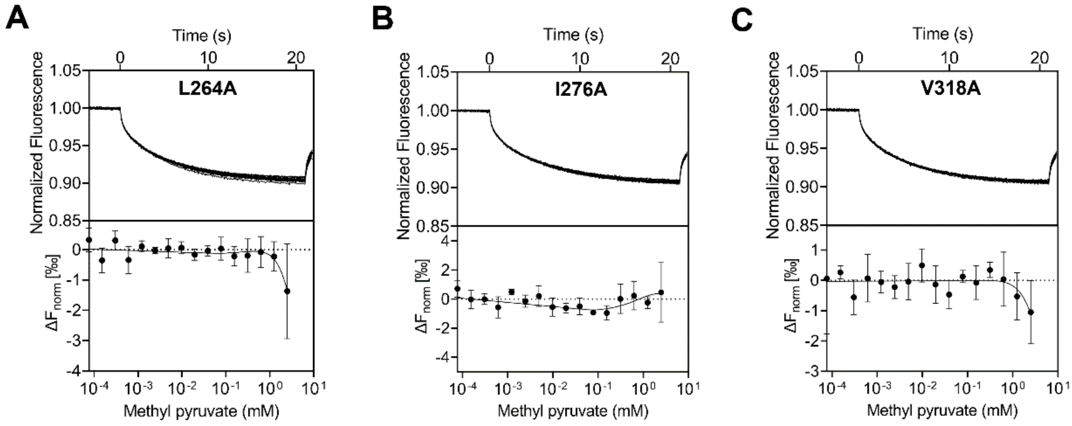

FIG S9 MST measurements for the interactions of Tlp11-LBD mutants with methyl pyruvate. (A–C) MST measurements for the interactions of Tlp11-LBD mutants L264A, I276A, and V318A with methyl pyruvate. The upper panel indicates the representative curves for thermophoresis of mutant proteins with different concentrations of methyl pyruvate, while the lower panel indicates the dose-response curve with the fitting result. Error bars represent the standard errors of three independent replicates, shown as mean ± SD. The concentration for the mutant proteins was 250 nM, and the maximum concentration for the ligand was 2.5 mM, which was diluted gradually.

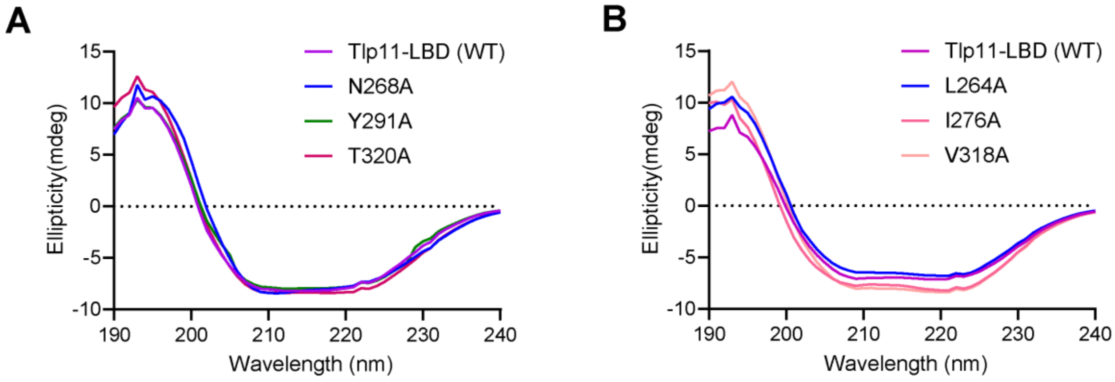

FIG S10 The results of CD spectroscopy for WT Tlp11-LBD and its mutants. (A) The CD spectroscopy detection for the Tlp11-LBD mutant proteins N268A, Y291A, and T320A. (B) The CD spectroscopy detection for the Tlp11-LBD mutant proteins L264A, I276A, and V318A. The spectrum for each protein was recorded at 25°C.

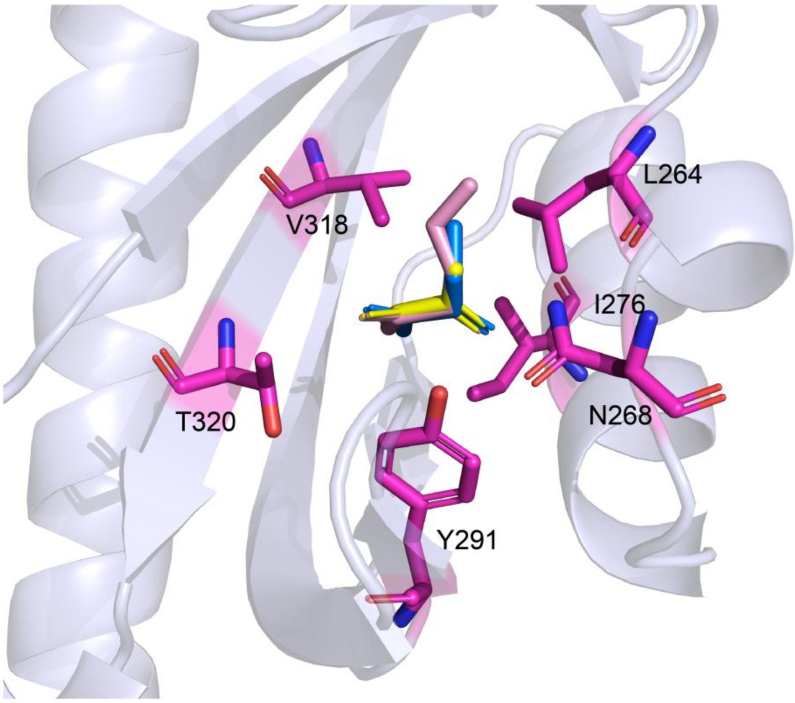

FIG S11 Molecular docking predictions of Tlp11 membrane-proximal subdomain binding to pyruvate, methyl pyruvate, and ethyl pyruvate. The interactions of Tlp11-LBD with pyruvate, methyl pyruvate and ethyl pyruvate were predicted by molecular docking and shown with PyMOL. The residues involved in methyl pyruvate binding in the membrane-proximal subdomain and their residue numbers are indicated. Pyruvate, methyl pyruvate and ethyl pyruvate are shown as yellow, marine and pink sticks, respectively.

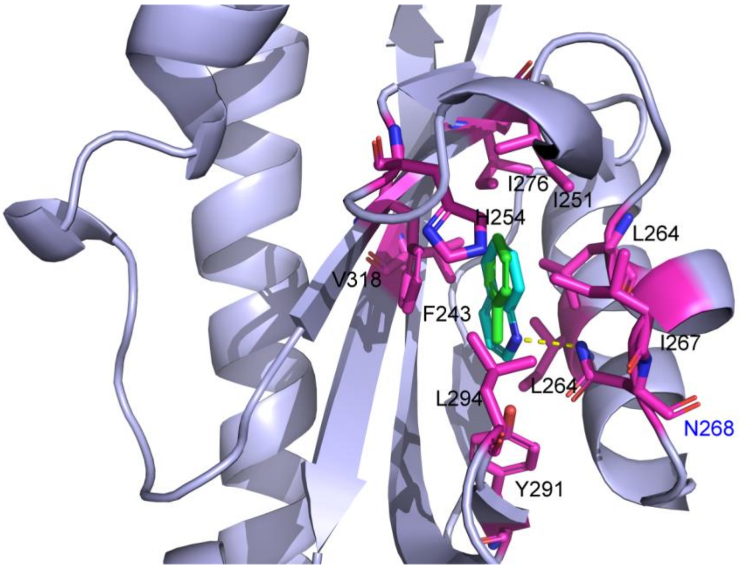

FIG S12 Molecular docking predictions of Tlp11 membrane-proximal pocket binding to toluene and quinoline. The interactions between Tlp11-LBD and toluene or quinoline were predicted by molecular docking and shown with PyMOL. The aromatic and non-polar residues in the membrane-proximal pocket and their residue numbers are indicated. Toluene and quinoline are shown as green and cyan sticks, respectively. The hydrogen bond is represented by yellow dashed lines.

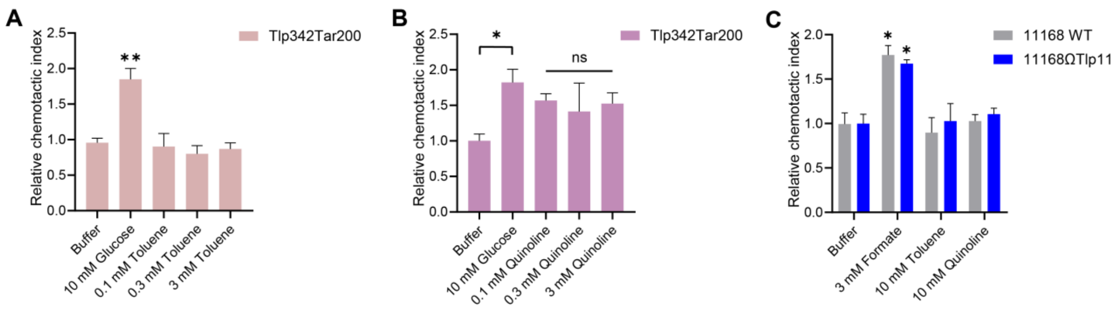

FIG S13 Chemotaxis of *E. coli* expressing hybrid receptor Tlp342Tar200 and *C. jejuni* to toluene and quinoline. (A,B) The relative chemotactic index of *E. coli* VS188 expressing Tlp342Tar200 in response to the indicated concentrations of toluene (A) and quinoline (B) at 50 min. (C) The relative chemotactic index of *C. jejuni* NCTC 11168 WT and 11168ΩTlp11 to toluene and quinoline at 30 min. The concentrations used for toluene and quinoline are shown in the figure. In (A−C), error bars indicate the standard errors of three independent replicates, shown as mean ± SD. The *p*-values were calculated using the paired *t*-test; *$p<0.05$, **$p<0.01$. In (B), “ns” means no significant difference among different quinoline concentrations.

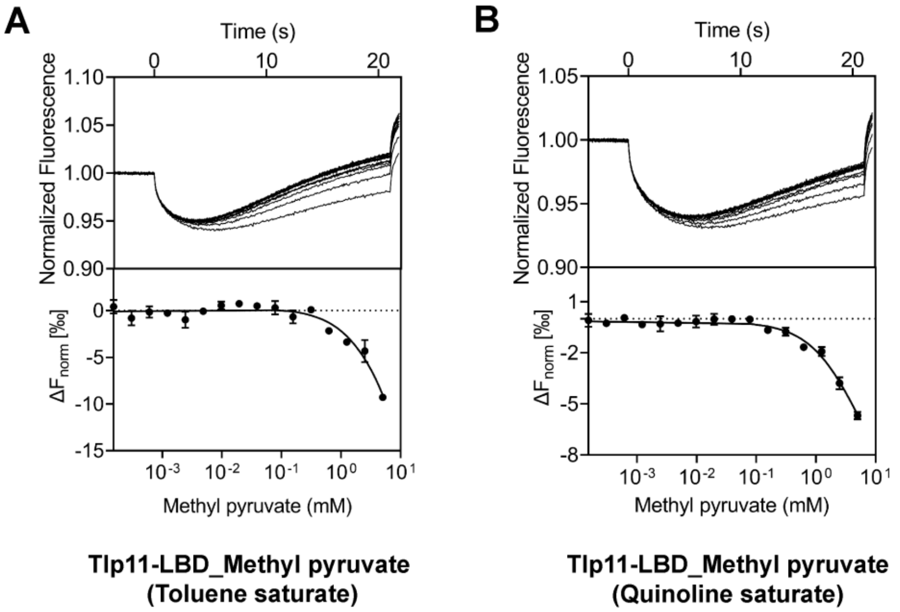

FIG S14 The effect of toluene and quinoline on the binding of Tlp11-LBD to methyl pyruvate. (A,B) MST measurements for the interactions of Tlp11-LBD with methyl pyruvate. The Tlp11-LBD was saturated with 5 mM toluene (A) or quinoline (B). The upper panel indicates the representative curves for thermophoresis of Tlp11-LBD after saturation with 5 mM toluene or quinoline with different concentrations of methyl pyruvate, while the lower panel indicates the dose-response curve with the fitting result. Error bars represent the standard errors of three independent replicates, shown as mean ± SD. The concentration for the Tlp11-LBD was 250 nM, and the maximum concentration for the ligand was 5 mM, which was gradually diluted.

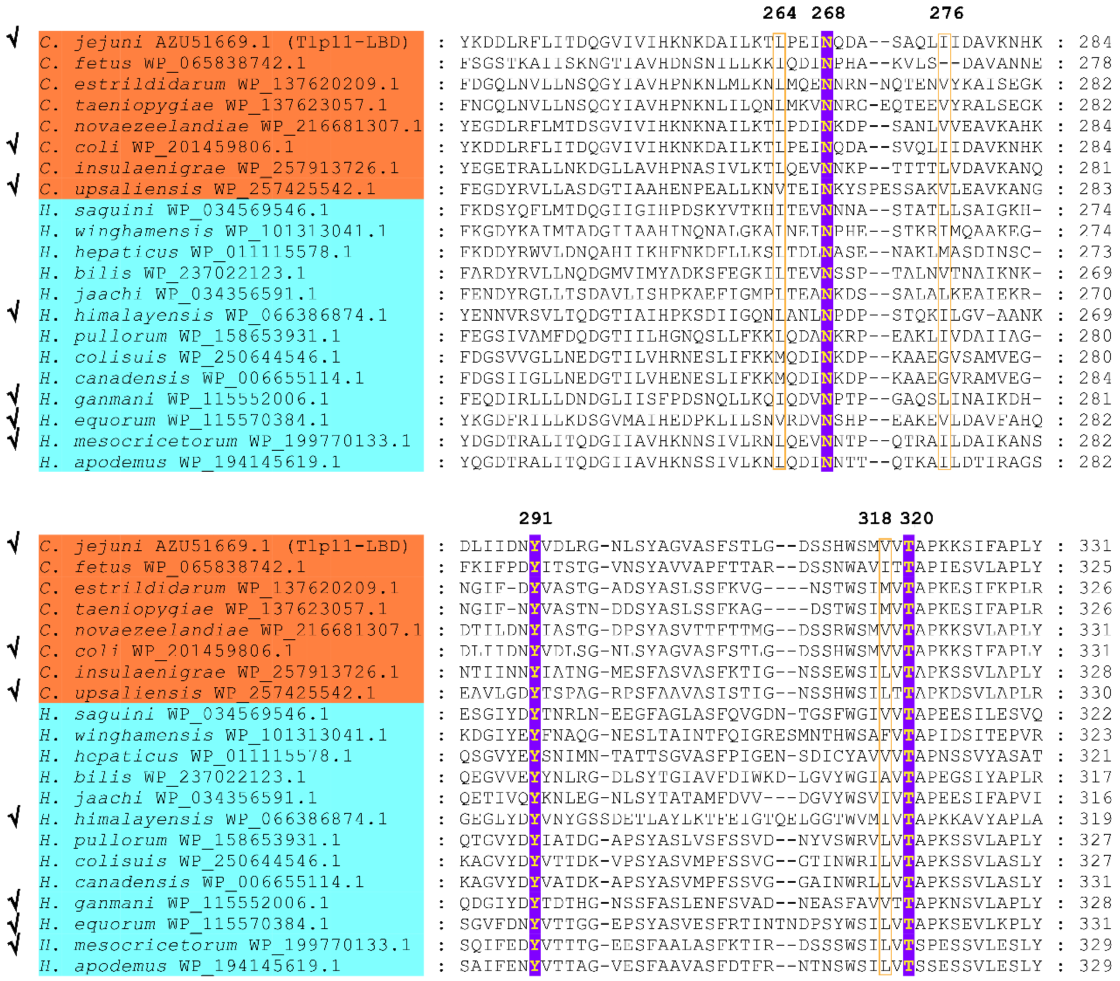

FIG S15 Sequence alignment for Tlp11-LBD homologues with the conserved residues N268, Y291, and T320. The NCBI accession numbers for Tlp11-LBD homologues from the species in genera *Campylobacter* and *Helicobacter* are shown in orange and cyan area, respectively. In sequence alignment, the purple area represents the key residues corresponding to Tlp11-N268Y291T320 that involved in hydrogen bond formation to methyl pyruvate. The residues that may be involved in hydrophobic interactions to methyl pyruvate are shown in yellow wireframes, and the black "√" indicates the sequences for experimental verification.

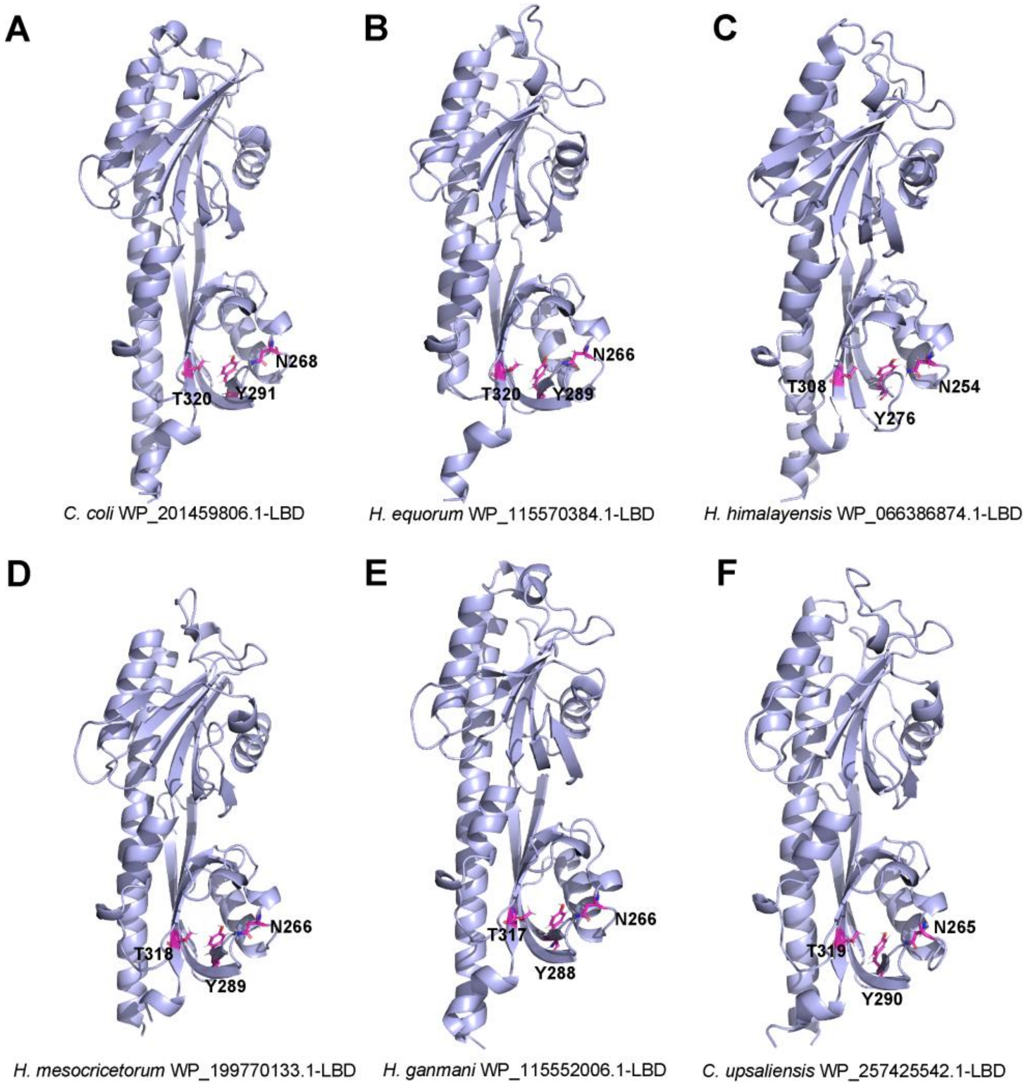

FIG S16 The predicted three-dimensional structures for Tlp11-LBD homologues. (A-F) The predicted LBD structures for Tlp11-LBD homologues in *C. coli*, *H. equorum*, *H. himalayensis*, *H. mesocricetorum*, *H. ganmani*, and *C. upsaliensis*. The key residues N, Y, and T involved in methyl pyruvate binding in the membrane-proximal pocket are displayed and labeled.

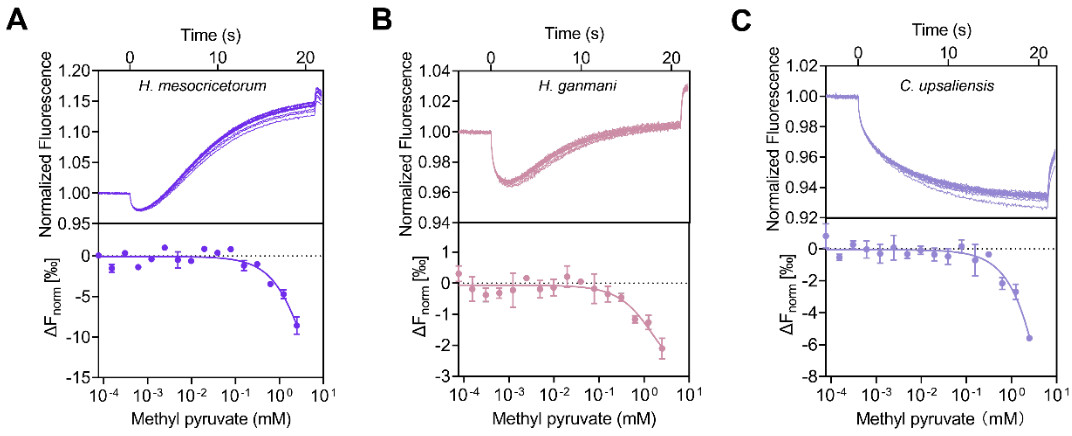

FIG S17 Microscale thermophoresis of methyl pyruvate towards Tlp11-LBD homologues. (A-C) The upper panel indicates the raw thermophoretic data, and the lower panel shows dose-response curves with fitting results. The working concentration of the proteins used for MST detection was 250 nM, and the maximum working concentration of the ligand was 2.5 mM, and it was gradually diluted. Error bars represent the standard errors of three independent replicates.
