# Supplementary material for: Identification of a dCache-type chemoreceptor in Campylobacter jejuni that specifically mediates chemotaxis towards methyl pyruvate
Source: Front Microbiol. 2024 May 9;15:1400284. doi: 10.3389/fmicb.2024.1400284 (PMC11111895; doi:10.3389/fmicb.2024.1400284)
Supplement: Supplementary file 2 [file Table_1.DOCX]

**Supplementary Tables**

**Table S1. Plasmids and bacterial strains used in this study**

| **Strains or plasmids** | **Description** | **Antibiotic resistance** | **Induction** | **Reference or source** |
| --- | --- | --- | --- | --- |
| **Plasmids** |  |  |  |  |
| pKG116 | Protein expression plasmid | Cm | - | (Burón-Barral et al., 2006) |
| pTrc99a-GFP | GFP expression plasmid | Amp | 50 μM IPTG | (Bi et al., 2016) |
| pUA66 | GFP reporter expression plasmid | Km | - | (Yuan et al., 2017) |
| pUA66-P*_mgtLA_*-GFP | GFP expression plasmid, *gfp* was placed under the *mgtLA* promoter, pUA66 derivative | Km | - | This study |
| pUA66-P*_ompC_*-GFP | GFP expression plasmid, *gfp* was placed under the *ompC* promoter, pUA66 derivative | Km | - | This study |
| pRE112 | Gene amplification plasmid | Cm | - | (Xi et al., 2019) |
| pBJ113 | Gene replacement vector containing *km* and *galk* cassettes | Km | - | (Zhuo et al., 2022) |
| pET28b | Protein expression plasmid | Km | - | Novagen |
| pZQ1 | Hybrid Tlp11[1-342]-Tar[200-553] expression plasmid, pKG116 derivative | Cm | 2 µM sodium salicylate | This study |
| pZQ2 | Hybrid Tlp11[1-343]-Tar[200-553] expression plasmid, pKG116 derivative | Cm | 2 µM sodium salicylate | This study |
| pZQ3 | Hybrid Tlp11[1-346]-Tar[204-553] expression plasmid, pKG116 derivative | Cm | 2 µM sodium salicylate | This study |
| pZQ4 | Hybrid Tlp11[1-347]-Tar[204-553] expression plasmid, pKG116 derivative | Cm | 2 µM sodium salicylate | This study |
| pZQ5 | Hybrid Tlp11[1-348]-Tar[204-553] expression plasmid, pKG116 derivative | Cm | 2 µM sodium salicylate | This study |
| pZQ6 | Tar expression plasmid, pKG116 derivative | Cm | 2 µM sodium salicylate | (Bi et al., 2016) |
| pPA791 | Pin-head Tar expression plasmid, pKG116 derivative | Cm | 2 µM sodium salicylate | (Bi et al., 2016) |
| pYFL1 | Hybrid kinase Tlp11[1-337]-PhoQ[200-486] expression plasmid, pKG116 derivative | Cm | 2 µM sodium salicylate | This study |
| pYFL2 | Hybrid kinase Tlp11[1-339]-PhoQ[200-486] expression plasmid, pKG116 derivative | Cm | 2 µM sodium salicylate | This study |
| pYFL3 | Hybrid kinase Tlp11[1-339]-PhoQ[201-486] expression plasmid, pKG116 derivative | Cm | 2 µM sodium salicylate | This study |
| pYFL4 | Hybrid kinase Tlp11[1-340]-PhoQ[202-486] expression plasmid, pKG116 derivative | Cm | 2 µM sodium salicylate | This study |
| pYFL5 | Hybrid kinase Tlp11[1-340]-PhoQ[200-486] expression plasmid, pKG116 derivative | Cm | 2 µM sodium salicylate | This study |
| pYFL6 | Hybrid kinase Tlp11[1-341]-PhoQ[202-486] expression plasmid, pKG116 derivative | Cm | 2 µM sodium salicylate | This study |
| pYFL7 | Hybrid kinase Tlp11[1-342]-PhoQ[202-486] expression plasmid, pKG116 derivative | Cm | 2 µM sodium salicylate | This study |
| pYFL8 | Hybrid kinase Tlp11[1-343]-PhoQ[202-486] expression plasmid, pKG116 derivative | Cm | 2 µM sodium salicylate | This study |
| pYFL9 | Full-length PhoQ expression plasmid, pKG116 derivative | Cm | 2 µM sodium salicylate | This study |
| pET28b-Tlp11-  LBD | Tlp11-LBD expression plasmid, pET-28b derivative | Km | 500 μM IPTG | This study |
| pET28b-Tlp11- LBD-N268A | Tlp11-LBD-N268A expression plasmid, pET-28b derivative | Km | 500 μM IPTG | This study |
| pET28b-Tlp11- LBD-Y291A | Tlp11-LBD-Y291A expression plasmid, pET-28b derivative | Km | 500 μM IPTG | This study |
| pET28b-Tlp11-  LBD-T320A | Tlp11-LBD-T320A expression plasmid, pET-28b derivative | Km | 500 μM IPTG | This study |
| pET28b-Tlp11-  LBD-L264A | Tlp11-LBD-L264A expression plasmid, pET-28b derivative | Km | 500 μM IPTG | This study |
| pET28b-Tlp11-  LBD-I276A | Tlp11-LBD-I276A expression plasmid, pET-28b derivative | Km | 500 μM IPTG | This study |
| pET28b-Tlp11-  LBD-V318A | Tlp11-LBD-V318A expression plasmid, pET-28b derivative | Km | 500 μM IPTG | This study |
| pET28b-  Tlp11-LBD homologue-*C. coli* | Tlp11-LBD homologue-  *C. coli* expression plasmid, pET-28b derivative | Km | 500 μM IPTG | This study |
| pET28b-  Tlp11-LBD homologue-  *H. equorum* | Tlp11-LBD homologue-  *H. equorum* expression plasmid, pET-28b derivative | Km | 500 μM IPTG | This study |
| pET28b-  Tlp11-LBD homologue-  *H*. *himalayensis* | Tlp11-LBD homologue-  *H*. *himalayensis* expression plasmid, pET-28b derivative | Km | 500 μM IPTG | This study |
| pET28b-  Tlp11-LBD homologue-  *H*. *mesocricetorum* | Tlp11-LBD homologue-  *H*. *mesocricetorum* expression plasmid, pET-28b derivative | Km | 500 μM IPTG | This study |
| pET28b-  Tlp11-LBD homologue-  *H*. *ganmani* | Tlp11-LBD homologue-  *H*. *ganmani* expression plasmid, pET-28b derivative | Km | 500 μM IPTG | This study |
| pET28b-  Tlp11-LBD homologue-  *C*. *upsaliensis* | Tlp11-LBD homologue-  *C*. *upsaliensis* expression plasmid, pET-28b derivative | Km | 500 μM IPTG | This study |
| pBJ114 | Gene replacement vector with *cm* and *sacB* cassettes, pBJ113 derivative | Cm | - | (Duan et al., 2023) |
| pTKRED | Helper plasmid for gene knockout, thermosensitive replicon | Spc | 500 μM IPTG | (Lange et al., 2019) |
| pBJ110-Ω*tlp11* | *tlp11* expression plasmid, pBJ114 derivative | Km | - | This study |
| pBJ110-Δ*cheY* | *cheY* knockout plasmid, pBJ114 derivative | Cm | - | This study |
| pRE112-Δ*phoQ* | *phoQup-tet-phoQdown* amplification plasmid, pRE112 derivative | Cm, Tet | - | This study |
| **Strains** |  |  |  |  |
| *E. coli* VS188 | Δ*aer*Δ*tsr*Δ*tar*Δ*tap*Δ*trg*, RP437 derivative | - | - | (Ames et al., 2002) |
| E. coli GB05-dir | (*fhu*A::IS2, Δ*ybcC*, Δ*recET*, P_BAD_-*ETγA*) with an arabinose inducible *ETγA* operon  (full-length *recE*, *recT*, *redγ* and *recA*), DH10B derivative | Sm | 17.5 mM  L-arabinose | (Wang et al., 2016) |
| E. coli GB08-red | (*fhu*A::IS2, Δ*ybcC*, Δ*recET*, P_BAD_-*γβαA*) with an arabinose-inducible *γβαA* operon (*redγ*, *redβ*, *redα* and *recA*), DH10B derivative | Sm | 17.5 mM  L-arabinose | (Wang et al., 2016) |
| E. coli BL21 (DE3) | F-ompT hsdSB(rB-mB-)gal dcm (DE3) | - | - | (Jeong et al., 2009) |
| *C. jejuni* NCTC 11168 | *C. jejuni* WT strain | - | - | (Marchant et al., 2002) |
| *C. jejuni* ATCC 33560 | *C. jejuni* WT strain | - | - | (McDermott et al., 2005) |
| *C. jejuni* NCTC 11168ΩTlp11 | tlp11 expressing strain in  C. jejuni 11168 | Km | - | This study |
| *C. jejuni* NCTC 11168/ΔCheY | cheY knockout strain in  *C. jejuni* NCTC 11168 | Km | - | (Duan et al., 2023) |
| *C. jejuni* NCTC 11168ΩTlp11/  ΔCheY | cheY knockout strain in  *C. jejuni* NCTC 11168ΩTlp11 | Km, Cm | - | This study |
| *E. coli* MG1655 | *E. coli* WT strain | - | - | (Baev et al., 2006) |
| *E. coli* MG1655-pTKRED | *E. coli* MG1655 containing the helper plasmid pTKRED | Spc | 500 μM IPTG | This study |
| *E. coli* MG1655/ΔPhoQ | phoQ knockout strain in  E. coli MG1655 | Tet | - | This study |

**Table S2 Compounds used for ligand screening of Tlp11-LBD in this study**

| **No.** | **Name** | **No.** | **Name** |
| --- | --- | --- | --- |
| 1 | L-Arabinose | 67 | D-Fructose-6-phosphate |
| 2 | N-Acetyl-D-glucosamine | 68 | α-Hydroxy glutaric acid-γ-lactone |
| 3 | D-Saccharic acid | 69 | α-Hydroxy butyric acid |
| 4 | Succinic acid | 70 | β-Methyl-D-glucoside |
| 5 | Niacinamide | 71 | Adonitol |
| 6 | L-Aspartic acid | 72 | Maltotriose |
| 7 | L-Proline | 73 | 2-Deoxy adenosine |
| 8 | D-Alanine | 74 | Adenosine |
| 9 | D-Trehalose | 75 | Glycyl-L-aspartic acid |
| 10 | D-Mannose | 76 | Citric acid |
| 11 | Dulcitol | 77 | m-Inositol |
| 12 | D-Serine | 78 | D-Threonine |
| 13 | D-Sorbitol | 79 | Fumaric acid |
| 14 | Glycerol | 80 | Bromo succinic acid |
| 15 | L-Fucose | 81 | Propionic acid |
| 16 | D-Glucuronic acid | 82 | Mucic acid |
| 17 | D-Gluconic acid | 83 | Glycolic acid |
| 18 | D,L-α-Glycerol-phosphate | 84 | Glyoxylic acid |
| 19 | D-Xylose | 85 | D-Cellobiose |
| 20 | L-Lactic acid | 86 | Inosine |
| 21 | Formic acid | 87 | Glycyl-L-glutamic acid |
| 22 | D-Mannitol | 88 | Tricarballylic acid |
| 23 | L-Glutamic acid | 89 | L-Serine |
| 24 | D-Glucose-6-phosphate | 90 | L-Threonine |
| 25 | D-Galactonic Acid-γ-lactone | 91 | L-Alanine |
| 26 | D,L-Malic acid | 92 | L-Alanyl-glycine |
| 27 | D-Ribose | 93 | Acetoacetic acid |
| 28 | L-Rhamnose | 94 | N-Acetyl-β-D-mannosamine |
| 29 | D-Fructose | 95 | Monomethyl succinate |
| 30 | Acetic acid | 96 | Methyl pyruvate |
| 31 | α-D-Glucose | 97 | D-Malic acid |
| 32 | Maltose | 98 | L-Malic acid |
| 33 | D-Melibiose | 99 | Glycyl-L-proline |
| 34 | Thymidine | 100 | p-Hydroxy phenyl acetic Acid |
| 35 | L-Asparagine | 101 | m-Hydroxy phenyl acetic acid |
| 36 | D-Aspartic acid | 102 | Tyramine |
| 37 | D-Glucosaminic acid | 103 | D-Psicose |
| 38 | 1,2-Propanediol | 104 | L-Lyxose |
| 39 | α-Keto-glutaric acid | 105 | Glucuronamide |
| 40 | α-Methyl-D-galactoside | 106 | Pyruvate |
| 41 | α-D-lactose | 107 | L-Galactonic acid-γ-lactone |
| 42 | Lactulose | 108 | D-Galacturonic acid |
| 43 | Sucrose | 109 | 4-Hydroxybenzoic acid |
| 44 | Uridine | 110 | Indoxyl acetate |
| 45 | L-Glutamine | 111 | D-Neopterin |
| 46 | m-Tartaric acid | 112 | 2-Nitropropane |
| 47 | D-Glucose-1-phosphate | 113 | Methyl formate |
| 48 | Uracil | 114 | Pyridoxine |
| 49 | Tyrosine hydrochloride | 115 | Melatonin |
| 50 | D-Valine | 116 | Levodopa |
| 51 | Acetoacetic acid | 117 | Norepinephrine |
| 52 | Taurine | 118 | Cinnamic acid |
| 53 | sec-Butylamine | 119 | Trimethylamine |
| 54 | Thymine | 120 | Hypoxanthine |
| 55 | Cytosine | 121 | Formamide |
| 56 | Purine | 122 | Serotonin |
| 57 | Acetic acid | 123 | Orotate |
| 58 | Butyrate | 124 | Dihydrouracil |
| 59 | Pyrimidine | 125 | Adrenaline |
| 60 | Adenine | 126 | Dopamine |
| 61 | Ethanolamine | 127 | Acetylcholine |
| 62 | Guanine | 128 | Thiazole |
| 63 | Xanthine | 129 | Amylamine |
| 64 | Uric acid | 130 | Pyridoxamine |
| 65 | Creatinine | 131 | 2-Aminooxazole |
| 66 | Tetrahydropyridine |  |  |

**Table S3. The analogues of methyl pyruvate for measurements**

| **No.** | **Name** | **Molecular formula** | **Structure** | **Molecular weight** |
| --- | --- | --- | --- | --- |
| 1 | Methyl pyruvate | C_4_H_6_O_3_ |  | 102.09 |
| 2 | Methyl formate | C_2_H_4_O_2_ |  | 60.05 |
| 3 | Methyl propionate | C_4_H_8_O_2_ |  | 88.11 |
| 4 | Ethyl pyruvate | C_5_H_8_O_3_ |  | 116.12 |
| 5 | Pyruvate | C_3_H_4_O_3_ |  | 88.06 |
| 6 | L-Methyl lactate | C_4_H_8_O_3_ |  | 104.11 |
| 7 | Methyl 3-hydroxybutyrate | C_5_H_10_O_3_ |  | 118.13 |
| 8 | Methylglyoxal | C_3_H_4_O_2_ |  | 72.06 |

**Table S4. The aromatic compounds for measurements**

| **No.** | **Name** | **Molecular formula** | **Structure** | **Molecular weight** |
| --- | --- | --- | --- | --- |
| 1 | O-Xylene | C_8_H_10_ |  | 106.17 |
| 2 | Benzoic acid | C_7_H_6_O_2_ |  | 122.12 |
| 3 | Benzotriazole | C_6_H_5_N_3_ |  | 119.13 |
| 4 | Benzimidazole | C_7_H_6_N_2_ |  | 118.14 |
| 5 | Phenol | C_6_H_6_O |  | 94.11 |
| 6 | Toluene | C_7_H_8_ |  | 92.14 |
| 7 | β-Phenylethylamine | C_8_H_11_N |  | 121.18 |
| 8 | Naphthoquinone | C_10_H_6_O_2_ |  | 158.16 |
| 9 | Naphthalene | C_10_H_8_ |  | 128.17 |
| 10 | Quinoline | C_9_H_7_N |  | 129.16 |
| 11 | Benzoquinoline | C_13_H_9_N |  | 179.22 |
| 12 | 4-Hydroxybenzoic acid | C_7_H_6_O_3_ |  | 138.12 |
| 13 | Menadione | C_11_H_8_O_2_ |  | 172.18 |
| 14 | L-Tyrosine | C_9_H_11_NO_3_ |  | 181.19 |
| 15 | L-Phenylalanine | C_9_H_11_NO_2_ |  | 165.19 |
| 16 | L-Tryptophan | C_11_H_12_N_2_O_2_ |  | 204.23 |

**Table S5. Distribution of chemoreceptor Tlp11 in *Campylobacter jejuni***

| ***C. jejuni* strains** | **Tlp11** | ***C. jejuni* strains** | **Tlp11** |
| --- | --- | --- | --- |
| NCTC 11168 | **-** | SHI5328 | **-** |
| NCTC 11351 | **+** | CJ512CC45 | **-** |
| ATCC 33560 | **+** | FJ3124 | **-** |
| CJ510CC45 | **-** | CJ074CC443 | **-** |
| NCTC 13265 | **-** | CJP19-D996 | **-** |
| HF5-4A-4 | **-** | FDAARGOS 422 | **-** |
| R19.1007 | **-** | AR-0419 | **-** |
| 1 | **-** | CJ518CC45 | **-** |
| NCTC 13268 | **+** | FORC 084 | **-** |
| CJ018CCUA | **-** | C57 | **-** |
| R18.1301 | **-** | 2016-IZSVE-19-111250 | **-** |
| G1 | **-** | C220 | **-** |
| FDAARGOS421 | **-** | C219 | **-** |
| RM1221 | **-** | C34 | **-** |
| YH002 | **+** | C197 | **-** |
| CP022076.1 | **-** | C41 | **-** |
| FDAARGOS262 | **+** | NCTC 12660 | **+** |
| ZP3204 | **-** | CS17 | **-** |
| TS1218 | **-** | CAMSA2002 | **+** |
| CFSAN032806 | **-** | WP2202 | **-** |
| MS2058 | **-** | CJ017CCUA | **-** |
| MS2005 | **-** | CC19PF065 | **-** |
| CS19 | **-** | CJ677CC520 | **-** |
| YQ2210 | **-** | CAMSA2038 | **+** |
| CJ071CC464 | **-** | CJ677CC032 | **-** |
| IF1100 | **-** | CJ677CC538 | **-** |
| YH003 | **-** | NS4-5-1 | **+** |
| CJ067CC45 | **-** | CJ001CC354 | **-** |
| AR-0414 | **-** | OD267 | **-** |
| PNUSAC004956 | **+** | CJ677CC073 | **-** |
| MS2167 | **-** | c021 | **-** |
| CJ015CC464 | **-** | CJ677CC014 | **-** |
| 12567 | **+** | C201 | **-** |
| CMP10201 | **-** | NCTC13255 | **-** |
| ZS005 | **-** | NCTC13261 | **+** |
| ZS007 | **-** | GB03 | **-** |
| CJ066CC508 | **-** | SHI6972 | **-** |
| CJ677CC533 | **-** | CJP19-D445 | **-** |
| CJ514CC45 | **-** | RM1477 | **-** |
| IA3902 | **+** | SHI3134 | **-** |
| 81116 | **-** | SHI4648 | **-** |
| 81-176 | **-** | CJ677CC531 | **-** |
| ATCC 35925 | **-** | ZS004 | **-** |
| RM1510 | **-** | CJ677CC036 | **-** |
| NS4-1-1 | **+** | NCTC13266 | **-** |
| CJ677CC002 | **-** | R12 | **-** |
| CJ677CC100 | **-** | CJ677CC024 | **-** |
| CJ677CC521 | **-** | CJ065CC48 | **+** |
| CJ677CC524 | **-** | CJ677CC523 | **-** |
| CJ677CC526 | **-** | CMP10190 | **-** |

“+” represents *C. jejuni* strain contains chemoreceptor Tlp11; “**-**” represents without chemoreceptor Tlp11. Universal primers (Fw: AGCAATAGGAATAGTCTTAGGCAT; Rv: GCACGAGCTGCTTCAATAGC) (Mund et al., 2016) for *tlp11* were used to evaluate whether Tlp11 exists. The 100 genomes from different *C. jejuni* strains in the NCBI Genome Database were randomly selected for analysis.

**Table S6. Tlp11-LBD homologous proteins**

| **Bacterial species** | **NCBI**  **accession number** | **Identity with**  **Tlp11-LBD sequence** |
| --- | --- | --- |
| *Campylobacter jejuni* | AZU51669.1 | 100% |
| *Campylobacter coli* | WP_201459806.1 | 98.67% |
| *Campylobacter novaezeelandiae* | WP_216681307.1 | 67.77% |
| *Campylobacter aviculae* | WP_137621885.1 | 41.86% |
| *Campylobacter insulaenigrae* | WP_257913726.1 | 40.07% |
| *Campylobacter upsaliensis* | WP_257425542.1 | 38.72% |
| *Campylobacter helveticus* | WP_257416057.1 | 37.75% |
| *Campylobacter vulpis* | WP_215728930.1 | 37.04% |
| *Campylobacter estrildidarum* | WP_137620209.1 | 37.25% |
| *Campylobacter taeniopygiae* | WP_137623057.1 | 36.58% |
| *Campylobacter fetus* | WP_065838742.1 | 34.67% |
| *Campylobacter hepaticus* | WP_066779406.1 | 34.01% |
| *Campylobacter avium* | WP_273404677.1 | 30.82% |
| *Campylobacter lanienae* | WP_086302056.1 | 26.33% |
| *Campylobacter volucris* | WP_151046648.1 | 26% |
| *Campylobacter troglodytis* | WP_142694466.1 | 26.64% |
| *Helicobacter mesocricetorum* | WP_199770133.1 | 37.15% |
| *Helicobacter apodemus* | WP_194145619.1 | 36.24% |
| *Helicobacter himalayensis* | WP_066386874.1 | 26.95% |
| *Helicobacter equorum* | WP_115570384.1 | 34.01% |
| *Helicobacter canis* | WP_210670680.1 | 30.46% |
| *Helicobacter pullorum* | WP_158653931.1 | 32.32% |
| *Helicobacter colisuis* | WP_250644546.1 | 31.54% |
| *Helicobacter didelphidarum* | WP_147290006.1 | 30.59% |
| *Helicobacter canadensis* | WP_006655114.1 | 32.12% |
| *Helicobacter bilis* | WP_237022123.1 | 27.30% |
| *Helicobacter trogontum* | WP_278871175.1 | 27.57% |
| *Helicobacter macacae* | WP_023928387.1 | 30.43% |
| *Helicobacter saguini* | WP_034569546.1 | 29.41% |
| *Helicobacter jaachi* | WP_034356591.1 | 27.33% |
| *Helicobacter rodentium* | WP_273211551.1 | 26.25% |
| *Helicobacter turcicus* | WP_221532466.1 | 29.80% |
| *Helicobacter ganmani* | WP_115551373.1 | 26.58% |
| *Helicobacter winghamensis* | WP_101313041.1 | 28.17% |
| *Helicobacter cinaedi* | WP_115721725.1 | 26.69% |
| *Helicobacter hepaticus* | WP_011115578.1 | 28.08% |
| *Helicobacter burdigaliensis* | WP_157978193.1 | 27.45% |
| *Helicobacter magdeburgensis* | WP_138128942.1 | 27.53% |
| *Helicobacter marmotae* | WP_147288786.1 | 25.16% |
| *Helicobacter felis* | WP_233704921.1 | 24.09% |
| *Wolinella succinogenes* | WP_011138770.1 | 25.49% |
| *Poseidonibacter lekithochrous* | WP_071625985.1 | 22.79% |
| *Candidatus Marinarcus aquaticus* | WP_128996723.1 | 25.97% |
| *Arcobacter* sp. F2176 | WP_129101968.1 | 26.88% |

**Supplementary References**

Ames P, Studdert C A, Reiser R H, Parkinson J S (2002). Collaborative signaling by mixed chemoreceptor teams in *Escherichia coli*. Proc Natl Acad Sci U S A, 99(10): 7060-7065

Baev M V, Baev D, Radek A J, Campbell J W (2006). Growth of *Escherichia coli* MG1655 on LB medium: determining metabolic strategy with transcriptional microarrays. Appl Microbiol Biotechnol, 71(3): 323-328

Bi S, Pollard A M, Yang Y, Jin F, Sourjik V (2016). Engineering hybrid chemotaxis receptors in bacteria. ACS Synth Biol, 5(9): 989-1001

Burón-Barral M C, Gosink K K, Parkinson J S (2006). Loss- and gain-of-function mutations in the F1-HAMP region of the *Escherichia coli* aerotaxis transducer Aer. J Bacteriol, 188(10): 3477-3486

Duan J, Zhao Q, Wang Y, Chi Z, Li W, Wang X, Liu S, Bi S (2023). The dCache domain of the chemoreceptor Tlp1 in *Campylobacter jejuni* binds and triggers chemotaxis toward formate. mBio, 14(3): e0356422

Jeong H, Barbe V, Lee C H, Vallenet D, Yu D S, Choi S H, Couloux A, Lee S W, Yoon S H, Cattolico L, Hur C G, Park H S, Ségurens B, Kim S C, Oh T K, Lenski R E, Studier F W, Daegelen P, Kim J F (2009). Genome sequences of *Escherichia coli* B strains REL606 and BL21(DE3). J Mol Biol, 394(4): 644-652

Jumper J, Evans R, Pritzel A, Green T, Figurnov M, Ronneberger O, Tunyasuvunakool K, Bates R, Žídek A, Potapenko A, Bridgland A, Meyer C, Kohl S a A, Ballard A J, Cowie A, Romera-Paredes B, Nikolov S, Jain R, Adler J, Back T, Petersen S, Reiman D, Clancy E, Zielinski M, Steinegger M, Pacholska M, Berghammer T, Bodenstein S, Silver D, Vinyals O, Senior A W, Kavukcuoglu K, Kohli P, Hassabis D (2021). Highly accurate protein structure prediction with AlphaFold. Nature, 596(7873): 583-589

Lange F, Pfennigwerth N, Höfken L M, Gatermann S G, Kaase M (2019). Characterization of mutations in *Escherichia coli* PBP2 leading to increased carbapenem MICs. J Antimicrob Chemother, 74(3): 571-576

Marchant J, Wren B, Ketley J (2002). Exploiting genome sequence: predictions for mechanisms of *Campylobacter* chemotaxis. Trends Microbiol, 10(4): 155-159

Mcdermott P F, Bodeis-Jones S M, Fritsche T R, Jones R N, Walker R D (2005). Broth microdilution susceptibility testing of *Campylobacter jejuni* and the determination of quality control ranges for fourteen antimicrobial agents. J Clin Microbiol, 43(12): 6136-6138

Mund N L, Masanta W O, Goldschmidt A M, Lugert R, Groß U, Zautner A E (2016). Association of *Campylobacter jejuni* ssp. *jejuni* chemotaxis receptor genes with multilocus sequence types and source of isolation. Eur J Microbiol Immunol (Bp), 6(3): 162-177

Wang H, Li Z, Jia R, Hou Y, Yin J, Bian X, Li A, Müller R, Stewart A F, Fu J, Zhang Y (2016). RecET direct cloning and Redαβ recombineering of biosynthetic gene clusters, large operons or single genes for heterologous expression. Nature Protocols, 11(7): 1175-1190

Xi D, Jing F, Liu Q, Cao B (2019). *Plesiomonas shigelloides sipD* mutant, generated by an efficient gene transfer system, is less invasive. J Microbiol Methods, 159: 75-80

Yuan J, Jin F, Glatter T, Sourjik V (2017). Osmosensing by the bacterial PhoQ/PhoP two-component system. Proc Natl Acad Sci U S A, 114(50): E10792-e10798

Zhuo L, Wan T Y, Pan Z, Wang J N, Sheng D H, Li Y Z (2022). A dual-functional orphan response regulator negatively controls the differential transcription of duplicate *groEL*s and plays a global regulatory role in *Myxococcus*. mSystems, 7(2): e0105621
